# Design of training populations for selective phenotyping in genomic prediction

**DOI:** 10.1038/s41598-018-38081-6

**Published:** 2019-02-05

**Authors:** Deniz Akdemir, Julio Isidro-Sánchez

**Affiliations:** 1 Cornell University Statistical Consulting Unit, Ithaca, NY USA; 2 School of Agriculture and Food Science, University College Dublin, Dublin, Ireland

## Abstract

Phenotyping is the current bottleneck in plant breeding, especially because next-generation sequencing has decreased genotyping cost more than 100.000 fold in the last 20 years. Therefore, the cost of phenotyping needs to be optimized within a breeding program. When designing the implementation of genomic selection scheme into the breeding cycle, breeders need to select the optimal method for (1) selecting training populations that maximize genomic prediction accuracy and (2) to reduce the cost of phenotyping while improving precision. In this article, we compared methods for selecting training populations under two scenarios: Firstly, when the objective is to select a training population set (TRS) to predict the remaining individuals from the same population (Untargeted), and secondly, when a test set (TS) is first defined and genotyped, and then the TRS is optimized specifically around the TS (Targeted). Our results show that optimization methods that include information from the test set (targeted) showed the highest accuracies, indicating that apriori information from the TS improves genomic predictions. In addition, predictive ability enhanced especially when population size was small which is a target to decrease phenotypic cost within breeding programs.

## Introduction

Genomic prediction (GP) uses high-density single nucleotide polymorphism markers across the whole genome to predict genetic values. This tool has been shown to be valuable in cases of animal and plant breeding, like in genomic selection (GS)^1^, disease risk predictions^2–4^ and personalized medicine^5,6^. Since its original formulation^1^, GS has shown better performance than traditional methods such as phenotypic selection, pedigree based selection and marker assisted selection^7,8^.

**Figure 1:**
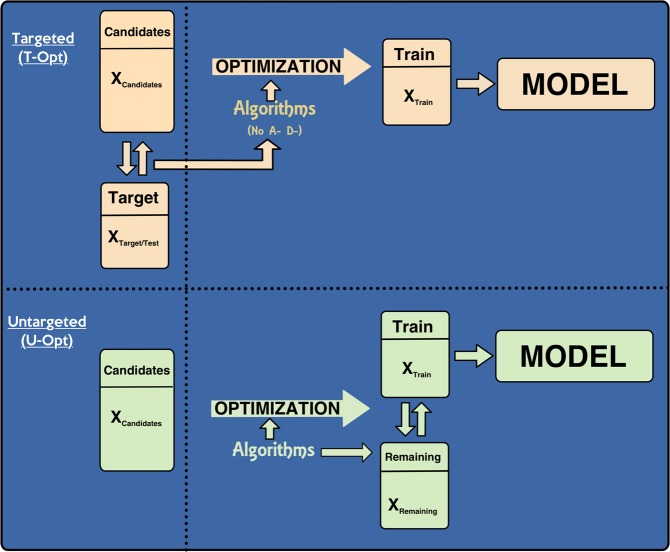
The two optimization schemes used in the article. The two scenarios in this figure are referred to as *U-Opt* and *T-Opt*: *U-Opt* describes the case where the data consists of the matrix of genomic scores for a candidate set, *X*_*Candidate*_; in *T-Opt*, we also know the genomic scores for a test set, *X*_*TS*_. Based on a design criterion, a training set of a given size is selected from the candidate set, the genomic score matrix for these individuals is denoted by *X*_*TRS*_. The genotypes in candidate set but not selected in the training set constitute the remaining set, with the score matrix *X*_*Remaining*_. We observe the phenotypes after the training set is identified and using these trait values in the training set and *X*_*TRS*_ a genomic prediction model will be built. The algorithms A- and D- Opt cannot use the genomic scores of the test set in *T-Opt*.

A critical step toward the implementation of GS is the establishment of the training population set (TRS). In GS, a TRS consisting of breeding lines phenotyped for target traits and genotyped with genome-wide markers is used to train a prediction model. Once is trained, this model is used to predict performance on a test set (TS) based solely on genotypic information by calculating genomic estimated breeding values (GEBVs) (Fig. 1). In this scheme, the prediction accuracy is estimated as the correlation between the GEBVs and the estimated genetic values. Comparisons among breeding methodologies are based on the prediction accuracy that is directly related to the breeders’ equation^9^. As such, prediction accuracy improvement is an important issue in GS applications. In this sense, many factors affect prediction accuracy^10^, including linkage disequilibrium (LD) between markers on TRS vs. TS^11,12^, genetic architecture^13–15^, statistical models^3,16^, heritability^17^, marker density^12,18^, population size^14,19^, and the genomic relationship between TRS and TS^20–27^.

The design of the TRS, also called optimization of the TRS, has attracted notable interest in the breeding community for several reasons. Firstly, the fact that predictions are based on markers or line effects calculated on the TRS raises the question of how to select the TRS to increase the efficiency and effectiveness of GS. Secondly, currently, the high cost of phenotyping makes the phenotype information the most important constraint in plant breeding programs. To better allocate resources within plant breeding programs the smaller the TRS the better, as they reduce phenotypic cost and increase the quality of the phenotypic data^19,28^. Thirdly, the traditional optimization process based on random sampling^29–31^ as a strategy to create the TRS does not always lead to an increase in predictive ability due to the under or over representation of the genetic information in the TRS^26,27,32,33^. Phenotyping has always been the bottleneck in breeding programs where progress from selection is directly correlated with the number of genotypes that could be evaluated phenotypically. In the genomic era, genotyping costs have dramatically dropped while the phenotyping costs stayed relatively constant. In this sense, one of the main aim of the optimization of the TRS is to improve the process of “selective phenotyping”, to reduce the cost of phenotyping while maintaining high prediction accuracy models.

In the last few years, different studies examined the importance of optimization of the TRS via comparing several sample selection approaches to random sampling with biparental populations^7,34–36^, with data combined cross multiple related and/or unrelated individuals^23–25,32^, and with diverse populations^26,27,37–39^. Two optimization criteria have been derived from the mixed model equations by Laloe^40^ and Rincent *et al*.^37^: the coefficient of determination (CD) and the prediction error variance (PEV). Rincent *et al*.^37^ concluded that maximizing CD was superior to PEV and random sampling because CD captured more genetic variability when selecting the individuals in TRS.

Recently, Isidro *et al*.^27^ proposed stratified sampling and stratified CD as alternative algorithms to improve the optimization of TRS under population structure effects. In this study^27^, concluded that the optimization of the TRS depended on the interaction of trait architecture and population structure, as well as on the ability of the algorithm to capture phenotypic variance. These authors^27^ also showed that stratified methods performed better when populations structure effects were pronounced.

One of the drawbacks of the aforementioned approaches is that they do not use information from the test set (TS) while building the TRS. This has been recently addressed by Akdemir *et al*.^26^ and Lorenz *et al*.^32^. Akdemir *et al*.^26^ used a genotypic algorithm to select optimized TRS using the genotypic information of the candidate and test individuals. This study showed that the information about the test genotypes leads to significant increases in accuracies, results that were also confirmed in Lorenz *et al*.^32^ who showed that adding unrelated individuals to the TRS can reduce the accuracy of the prediction models. The implementation of this TRS optimization methodology improved the performance of GP models. Recently, Bustos-Korts *et al*.^33^, proposed another TRS construction method that sample the genetic space uniformly, to increase predictive ability under mild population structure effects. The results of their methods were similar to those of previous methods in the sense that optimized selection strategies resulted in increases in accuracies as compared to random training samples.

The optimal design of TRS for GP is essentially an optimal experimental design problem that has always captured the attention of many statisticians in the past^41–48^ but remains relatively unexamined in the breeding community. The concept of Design of Experiments (DOE), should be used to plan experimental designs and perform sets of well-selected optimization TRS to get the most informative combination out of the given factors.

Given the importance of the DOE for effective implementation of optimization of the TRS, the objective of this paper was (1) to add DOE approaches to TRS optimization and (2) to compare several DOE strategies under two breeding scenarios. The first scenario, “Untargeted optimization” (U-Opt), when the information of the TS is not used to build the TRS model, and the second scenario, “Targeted Optimization” (T-Opt), when using the information from the TS to build the TRS (Fig. 1).

## Results

**Figure 2:**
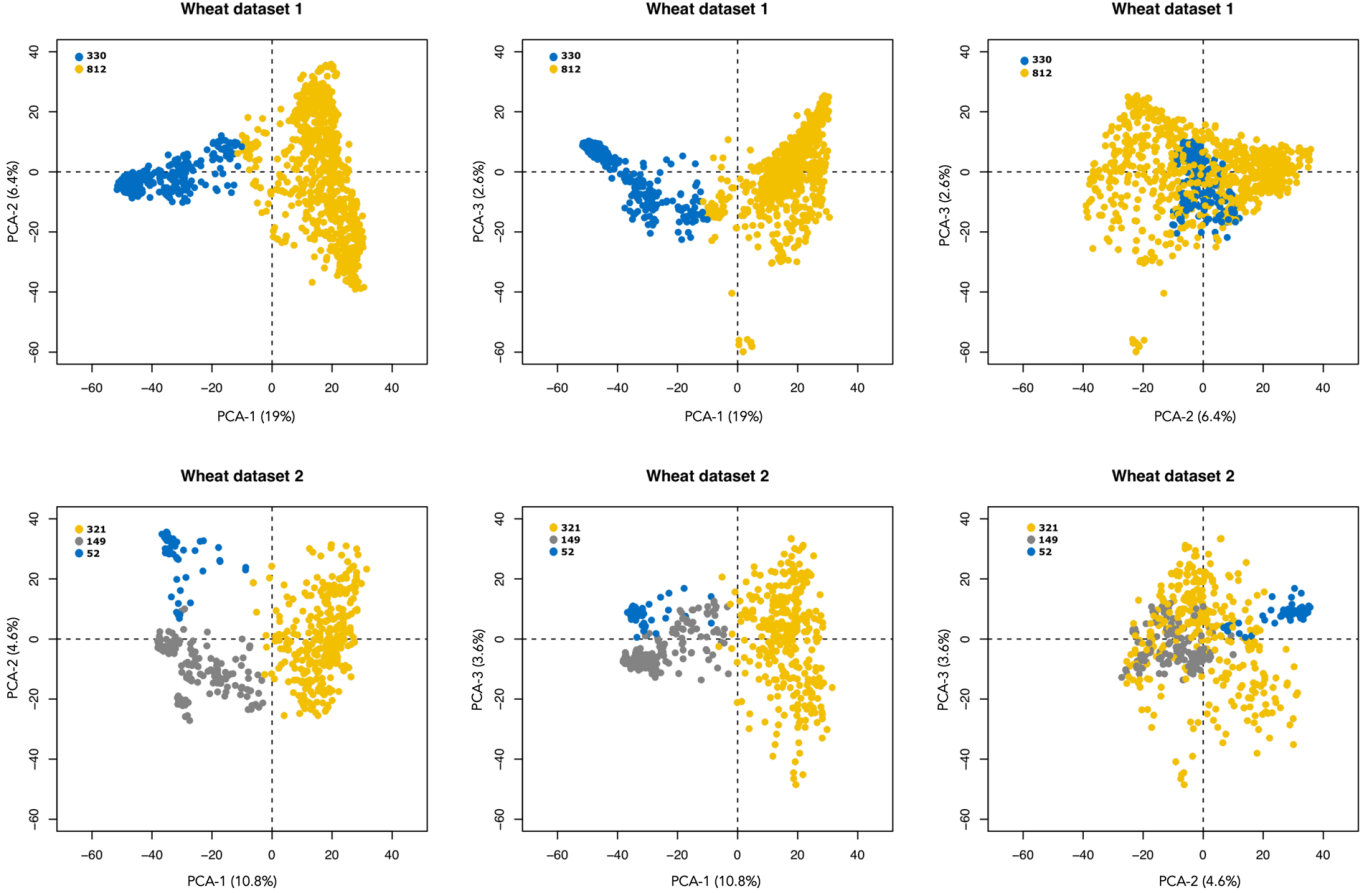
Plots of the three first principal components and the cluster analysis using dataset 1 and 2. Each solid circle represents a genotype and the colors indicate cluster membership. Number of genotypes per cluster are given by the figure legends.

**Figure 3:**
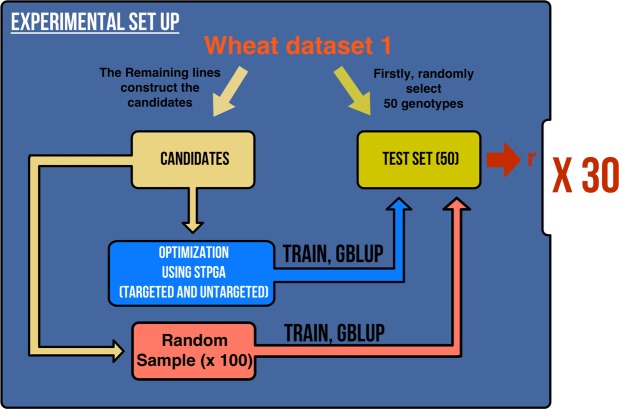
Example of the experimental set up optimization scheme applied to the wheat dataset 1. From the base population, a random sample of 50 genotypes was selected to build the test set. The remaining genotypes formed the candidates set were the optimization process takes places. From these candidates, four different algorithms using STPGA were used to build the training population set (TRS) test population size under two scenarios (Untargeted-Opt and Targeted-Opt). At the same time, a random sample of the same size was selected as the TRS. This process was repeated 100 runs. The entire set was repeated 30 times. Prediction accuracies (r) were calculated using GBLUP method.

Briefly, the genetic material used in this study consists of two wheat datasets downloaded from triticeae toolboox database. Wheat dataset 1 consists of 1693 lines evaluated for six traits in three environments after data curation. Wheat dataset 2 consisted of 520 genotypes evaluated on seven traits and 3 environments after data curation. Principal components analysis (PCA) on marker data was used to visualize the structure of the populations (Fig. 2). For both datasets, missing marker data were imputed using a multivariate normal expectation maximization (EM) algorithm. The optimization methodologies described in this manuscript were implemented with the R package “Selection of the Training Populations with a Genetic Algorithm” (STPGA)^49^. The optimization schemes are shown in Fig. 1 and the experimental setup in Fig. 3.

### Population structure

The population structure (PCA) analysis on a 3D-scatter plot is displayed in Fig. 2. In dataset 1, the first three principal components (PCs) accounted for 19%, 6.4% and 2.6% of the genetic variance. In the dataset 2, the first 3 PCs accounted for 10.8%, 4.6% and 3.6%. These results indicate that both datasets showed mild population structure. In Fig. 2, we also indicated the clustering that best represents the structure in these datasets by plotting genotypes in different clusters and colors. Population sizes within clusters varied from 812 to 330 genotypes on dataset 1 and from 321 to 52 in dataset 2.

Some individuals are frequently selected to be in the optimized training populations. The frequency of selection information for different optimization methods are displayed in PCA plots in the Supporting Information (S1 and S2). These graphs can give insight into how the different optimization methods differ in their behaviors. For example, CD_*mean*_ obtained a better representation of the genetic space than PEV_*mean*_ for dataset 2. CD_*mean*_ optimization has obtained better representation of the genetic space compared to the PEV_*mean*_ for the mixed model formulation based on GBLUP.

### Prediction accuracies

**Figure 4:**
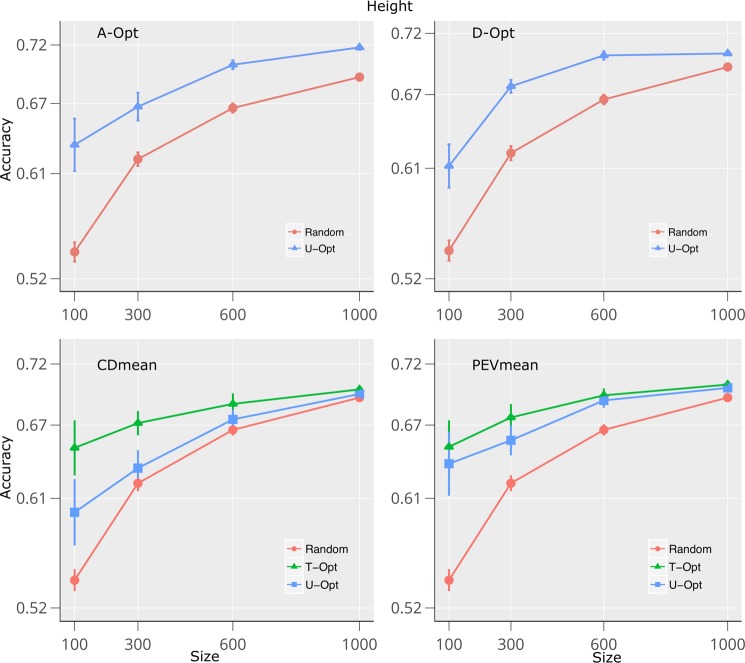
Prediction accuracies for height trait using sampling algorithms within STPGA package on dataset 1. Accuracies of the predictions of the test set (TS) genotypes were calculated using 4 different algorithms and 2 methods compared with random sampling. In the *U-Opt* method, the TS were not used to build the training population set (TRS) while in the *T-Opt* the optimization algorithm used the TS to build the TRS. The TRS were defined by optimizing A-Opt, D-Opt, CD_*mean*_, and PEV_*mean*_. Four different population sizes (100, 300, 600 and 1000) were used for the optimization algorithm. Standard error is indicated for each point over 30 (*U-Opt* and *T-Opt*) and 100 (*random*) runs.

**Figure 5:**
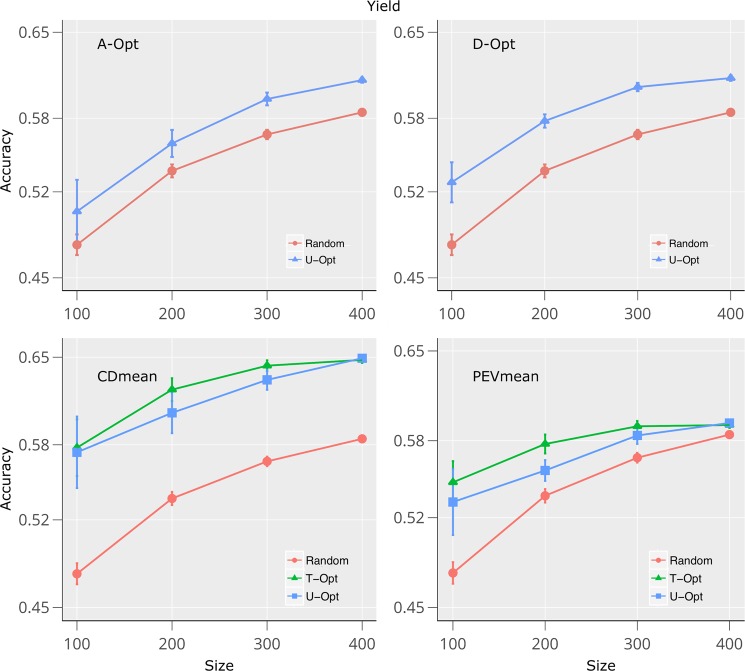
Prediction accuracies for yield using sampling algorithms within STPGA package on dataset 2. Accuracies of the predictions of the test set (TS) genotypes were calculated using 4 different algorithms and 2 methods compared with random sampling. In the *U-Opt* method, the TS were not used to build the training population set (TRS) while in the *T-Opt* the optimization algorithm used the TS to build the TRS. The TRS were defined by optimizing A-Opt, D-Opt, CD_*mean*_, and PEV_*mean*_. Four different population sizes (100, 200, 300 and 400) were used for the optimization algorithm. Standard error is indicated for each point over 30 (*U-Opt* and *T-Opt*) and 100 (*random*) runs.

Figures 4 and 5 show the accuracies of the four criteria analyzed in this study for the traits plant height in dataset 1, and grain yield in the dataset 2. The complete set of traits are shown in Supplementary Figs (S3–S13). In both datasets, the accuracy of GEBVs increased and the sampling variance decreased as TRS size populations increased. Predictions that used the TS to build the TRS (T-Opt) had higher accuracies than the other scenarios (U-Opt and random) for both datasets and all traits. On average, random samples had the lowest accuracies across datasets and traits, especially at the lowest TRS size Figs 4, 5 and Supplementary Figs (S3–S13).

#### Wheat dataset 1

For the sake of simplicity, we only display the results for height in the main text. The results for the complete set of traits is shown in Supplementary Figs (S3–S7).

Across all traits, accuracies ranged from 0.45 to 0.79 Adult rust severity-T1 showed the highest accuracy across traits, followed by height, adult rust severity, adult reaction type T1, heading date and adult rust reaction. The smaller the TRS sizes, the larger were the differences between random sampling and optimization criteria, especially when using the algorithms CD_*mean*_ and PEV_*mean*_. This trend was also observed under A-opt and D-opt but the differences were smaller, especially in heading, adult rust reaction type and adult rust severity T1 (S3, S5, S8). Our results also showed that the best accuracies were reached when the TS information was included in building the TRS. Figure 4 and Table 1 show the accuracies of the predictions for height for the dataset 1. The accuracies ranged from 0.72 to 0.53. Maximum accuracies were obtained with the largest TRS size, although the differences between the lowest and the largest TRS size (9 folds of difference) were less than 16% on average across optimization methods. If we compare 1000 vs 300 TRS, then the differences drop to less than 8%. As the general trend indicates, optimized samples (*U-Opt*, *T-Opt*) showed a 1.5% greater accuracy than random sampling with a maximum improvement of 3.1% at 100 TRS size.

**Table 1:** Relative prediction accuracy of height within the dataset 1 using the random sample as reference.

Size	Test	A-opt	D-opt	CD_*mean*_	PEV_*mean*_
100	Random	100.0	100.0	100.0	100.0
	U-Opt	111.8	112.1	112.2	115.8
	T-Opt	—	—	122.1	118.3
300	Random	100.0	100.0	100.0	100.0
	U-Opt	103.1	108.2	102.5	104.7
	T-Opt	—	—	108.5	107.7
600	Random	100.0	100.0	100.0	100.0
	U-Opt	101.8	104.6	101.6	102.9
	T-Opt	—	—	103.5	103.5
1000	Random	100.0	100.0	100.0	100.0
	U-Opt	100.3	100.8	100.5	100.4
	T-Opt	—	—	101.0	100.8

#### Wheat dataset 2

Across all traits, accuracies ranged from 0.86 to 0.28 (S8–S13). Lodging and lodging 2 showed the maximum accuracies, with the lowest observed via test weight. Both optimization criteria (*U-Opt and T-Opt*) had, on average, 4% greater accuracies than random sampling among all traits and TRS sizes. As in dataset 2, the smaller the TRS size the larger differences between random sampling and the optimization criteria, especially when using the algorithms CD_*mean*_ and PEV_*mean*_. Accuracies increased as TRS size increase for all traits, except in test weight for D-opt and PEV_*mean*_.

On average among the two methods (*U-Opt and T-Opt*), best accuracies were reached when TS information was included in building the TRS (T-Opt). Among optimization methods, CD_*mean*_ and PEV_*mean*_ had 0.7% greater accuracies than A-opt and D-opt.

Figure 5 and Table 2 show the accuracies of the predictions for grain yield in dataset 2. The accuracies ranged from 0.65 to 0.48 Maximum accuracies were obtained with the largest TRS size, although the differences between the lowest and the largest TRS size were less than 15%, on average across optimization methods. If we compare 500 versus 300 TRS, then the differences drop to less than 6.5%. As the general trend indicates, the optimization criteria (*U-Opt*, *T-Opt*) showed a 4.7% greater accuracy than random sampling across optimization methods and TRS size, with a maximum improvement of 14.1% at 100 TRS size.

**Table 2:** Relative prediction accuracy of yield within the dataset 2 using the random sample reference.

Yield	Train	A-opt	D-opt	CD_*mean*_	PEV_*mean*_
100	Random	100.0	100.0	100.0	100.0
	U-Opt	103.8	107.1	111.3	110.9
	T-Opt	—	—	112.0	114.1
200	Random	100.0	100.0	100.0	100.0
	U-Opt	102.5	104.4	103.7	103.1
	T-Opt	—	—	106.9	107.0
300	Random	100.0	100.0	100.0	100.0
	U-Opt	102.7	104.0	102.6	102.8
	T-Opt	—	—	104.5	104.1
400	Random	100.0	100.0	100.0	100.0
	U-Opt	101.8	102.0	102.2	101.6
	T-Opt	—	—	102.0	101.3

## Discussion

In the last few years, the optimization of the TRS have been addressed in several studies^22,25–27,32,33,37,50–52^. These studies have helped researchers better understand the factors affecting TRS optimization and core subset selection. In this paper, we compared the performance of four optimization methods under two scenarios (*U-Opt* and *T-Opt*) on two datasets and 13 traits. In addition, we compare several designs of experiment strategies to define an optimal TRS using a genetic algorithm. Our results indicated that the optimization approach showed better predictions on average than did random sampling. We found the smaller the sample size, the higher the benefits obtained from an optimized TRS. We also showed that the procedure *T-Opt* was consistently greater than *U-Opt* which indicates that the use of TS information while building the TRS plays a critical role in improving accuracies (Figs 4 and 5)^26,32^. While the ordering of the different criteria did not change when increasing TRS, the differences among criterion decreased. The predictive ability enhanced when population size is small which is a key target to decrease phenotypic cost within breeding programs.

Dataset 1 showed the smallest difference between random sampling and the optimization methods. In fact, some traits, such as reaction time Type 1, reaction time and adult rust severity T1 showed better accuracies especially under A-opt and D-opt algorithm methods (S3–S6). This seems to endorse previous results^27,33,53^ where genetic architecture (GA) and population structure (PS) play key roles in improving model performance and TRS design. In fact, dataset 1 showed higher PS effects than dataset 2 and traits were rather qualitative than quantitative. The differences in methods were larger in regards to traits governed by a higher number of genes with small effects, such as height and yield (Figs 4 and 5), which indicates that selection methodologies in this paper worked better with complex traits. It is important to note that, the larger accuracies found using STPGA algorithm is not a GA and PS artefact since on average the algorithm showed greater accuracies than random sampling among datasets (Tables 1 and 2). These results suggest that, if the population under study is highly structured, then applying a stratified sampling scheme–which might involve sampling optimal sets within each strata–might be more appropriate^27^. The importance here is that our optimized methods can be applied to multiple breeding contexts from one breeding cycle where all data is genotyped to multicycle where data from previous cycles is available to predict the current cycle.

The experimental scenario under *T-Opt* involves knowing the genotypic information about the TS of individuals. The design criteria PEV_*mean*_ and CD_*mean*_ can make use of this information, resulting in higher accuracies for each sample size. The design criteria A-opt and D-opt do not use the test information and the accuracies of these criteria are the same under the two scenarios *U-Opt* and *T-Opt*. Nevertheless, design criteria such as A-opt or D-opt are viable alternatives to the PEV_*mean*_ and CD_*mean*_ from the DOE framework perspective especially when the number of genes controlling the trait is low, since A-opt and D-opt are computationally more efficient than PEV_*mean*_ or CD_*mean*_. The readers can refer to the STPGA R Package help files and vignette to implement a larger number of others DOE approaches.

In order to evaluate how much accuracy is sacrificed using the approximations based on the use of principal components, we have devised an experiment where the results obtained using approximations were compared to those obtained using the full data (using the formulas in Equations 11 and 12 for genomic relationship matrices). In this experiment, the accuracies of optimal sets obtained using criteria 11, 12 were compared to their equivalents calculated using the 10, 50, and 100 PCs based on accuracies evaluated over several traits from two wheat datasets. The results are summarized with the Supplementary Figs (S14 and S15). According to these results, 10 PCs were not sufficient for the optimization of the training populations since the samples obtained using 10 PCs on average performed similarly to the random samples. The accuracies of samples obtained using the full data methods showed a slight improvement on average compared to the samples obtained using 50 or 100 PCs. Therefore, the full data approach might be preferred when the computational resources are sufficient. Nevertheless, when our approximation is used the number of principal components are chosen such that a considerable amount of genetic variation is captured.

Based on the reviewers’ suggestions, we have also added results from an experiment where the target population size was changed to see the effect of this component in accuracies. In this experiment, the accuracies of optimal sets obtained using A-opt, D-opt, CD_*mean*_, PEV_*mean*_ and the targeted versions of CD_*mean*_, PEV_*mean*_ were compared based on accuracies evaluated over several traits for Dataset 1 for randomly selected target populations of sizes 50, 100, 200 and 400 and a fixed TRS of 100 and 300. The results are summarized with the Supplementary Figs S16 and S17. Based on the results of this experiment, the benefits of selecting a targeted population decreased as the target population size increased; for large samples, the performance of targeted populations was similar to untargeted populations. In general, optimized training populations gave better accuracies than the random training populations for all target population sizes. The decrease in the difference of accuracies between the targeted and untargeted training populations as the target size increases is expected. This is since optimization for a small set of randomly selected individuals from a genetic population will be more specific than optimization for a large set of randomly selected individuals from the same population. Nevertheless, as was observed in^26^, targeted optimization is also useful in scenarios where the candidate set and the target set are coming from different genetic populations. For instance, the target population might be the breeding population in the current year and the candidate population can be the previous genetic populations that gave rise to the current population. The benefits of using a targeted population are expected to be greater when the candidate set and target sets represent different parts of the genomic space.

The selection of TRS will become more important as more genotypic and phenotypic information is accumulated in breeding databases, such as zeabase (http://www.plantgdb.org/ZmGDB/), triticeae toolbox (https://triticeaetoolbox.org), CIMMYT (http://data.cimmyt.org/dvn/), solcap (http://solcap.msu.edu/tomato_genotype_data.shtml), rice diversity (https://ricediversity.org/), etc… Such databases are the results of collaborative efforts of breeders through years, and as such contain valuable information. For example, using the techniques in this article, we can identify a set of genotypes that have trait records relevant to a current breeding population, and supplement our experimental data with relevant data from a public database. This might even allow for the building of prediction models for a breeding population without doing any phenotypic experiment within that population. Such a prediction model would be built by merely using the relevant data from the database. A related application of TRS is toward defining a representative sample of genotypes in a seedbank, which we refer to as a core population, for characterization of the phenotypes that correspond to all genotypes in the seedbank. The phenotypic experiments can then be run only on this optimized core set to build models, and these models could then be used to estimate phenotypes. This approach was shown to be promising by Crossa *et al*.^54^.

Most of the plant breeding efforts concentrate on improving the trait values at each cycle. With GS, the improvement in trait value is related to the accuracy of the GEBVs, and this, in turn, depends on the quality of the TRS of genotypes that are used in these models. In general, it is possible to imagine cases where the TRS is different from the breeding population and can be designed optimally without changing it. However, in many cases, these two populations coincide to some extent, as the same genotypes are used for training models and for making decisions about forming the next progeny to improve trait values. In such cases, a breeding strategy can be used to balance the two goals simultaneously, improving trait values and at the same time making sure the next generation also makes a good TRS. These are competing goals and, as in any multiobjective optimization problem with competing objectives, will define a frontier of non-dominated (Pareto optimal) solutions^55,56^.

The use of optimization algorithms can also be implemented within the mating plan phase on plant breeding programs. For example, if we want to increase the genomic predictability of a breeding population while also increasing gains, we could select mates in each round to optimize a weighted sum of expected gains while improving the predictability of the TRS. In this sense, the design criterion is calculated using the expected genotypic values for the progeny according to the given mating plan. The authors of this article have also published work that suggests using optimal genomic mating plans for the improvement of breeding populations in the long run^55^. The mating approach introduced in that paper or selection based breeding can be used for optimizing other quantities of interest. Similar strategies can be used for designing and improving association mapping populations. We aim to address these issues with a subsequent article. Finally, if the test population is different from the one that is targeted, the advantages of the targeted approaches against the untargeted approaches disappear. Nevertheless, optimized procedures including the targeted ones still retain the advantage over random samples of the same sizes. An experiment was run to demonstrate this and the results are displayed with Supplementary Figs S18–S21.

With the experiments presented in this paper, we have shown that, given a TS, it is possible to predict them more accurately compared to a random sample of the same size. Based on this, it seems plausible to define genomic prediction models dynamically by using only a subset of the available phenotype and genotype data. Taking this idea to the extreme, we could say that a core subset of observations in the dataset are more relevant to this individual, and these should be the only individuals to be used for building an individual-specific model. Note that this problem is related to the design problem studied in this paper, but the dynamic modeling involves a dataset complete with phenotypic and genotypic records. Authors of this paper have tried to fit dynamical models to a variety of genomic datasets and observed no significant changes in results compared to the models that use the totality of the data. Nevertheless, this topic could be explored further, possibly via genomic datasets with different degrees of family structure.

Finally, there have been many approaches that used a single selection criteria for designing genomic prediction training populations with the promise of improving genomic prediction accuracies and therefore improving expected gains from GS^26,27,37^. The list of optimization criteria has been extending and most of the literature is devoted to demonstrating the advantages and disadvantages of using one of these methods over the other. In our opinion, this debate can be partially circumvented by designing training populations that are optimal for multiple design criteria at the same time, in the multi-objective optimization framework. For example, a multi-objective optimized training population selection approach might seek solutions that balances genomic diversity in the training population, genetic closeness to a target population (the GS model trained in the training population will be used to predict GEBVs in the target population) in addition to some other criteria related to selection of training populations. The advantages to using more than one criterion to design experiments are twofold. Firstly, the study of the trade-offs between the different design criteria aids in the decision about the weights that can be assigned to each of the criterion for a given experiment and therefore by-passing the question about which criterion to choose. Secondly, the relationships and trade-offs among the criteria over different experiments can help us understand their behavior with respect to each other and therefore aid in the choice of the set of criteria to be considered for a particular design problem the first place. The latest version of the STPGA package has many design criteria that can be used to find optimal training populations.

## Materials and Methods

### Materials

The genetic material used in this study consists of two wheat datasets downloaded from triticeae toolboox database (triticeaetoolbox.org/). The main characteristics of these datasets are described in Table 3. The data belongs to the data program of the national small grains collection.

**Table 3:** Germplasm description summary for dataset 1 and 2.

Dataset	Lines	Markers	#Environments	Traits
Dataset 1	1693	4670	3	Plant height
				Adult rust severity
				Adult rust severity T1
				Adult rust Reaction
				Adult rust Reaction type I
				Heading date
Dataset 2	528	5074	3	Plant height
				Test Weight
				Lodging
				Test Weight
				Yield
				Grain protein
				Heading date (Julian)

For both datasets, missing marker data were imputed using a multivariate normal (MVN)- expectation maximization (EM) algorithm^57^. The EM algorithm represents a general approach to calculating maximum likelihood estimates of unknown parameters when data are missing^58^.

Principal components analysis on marker data was used to visualize the structure of the populations.

Marker data were used to calculate the Euclidean distances between genotypes, and hierarchical clustering analysis using the Ward criterion was applied to the Euclidean distance matrix (Fig. 2). From the screeplots analysis, we selected 2 clusters for dataset 1 and 3 for dataset 2.

We have used a mixed model that considers the effects of environments as fixed effects and the genomic values (GVs) as random effects in a mixed model; in this model, the GVs were assumed to be distributed independent and identically with a zero-centered normal distribution. Estimated GVs were used in the subsequent analysis, for model building and evaluation of accuracies.

#### Wheat dataset 1

Phenotypic data for six traits were analyzed in this dataset: plant height (cm), adult stripe rust severity (%), adult stripe rust reaction type (rating 0–9), adult stripe rust reaction type T1 (rating 0–9), stripe rust severity (%) and stripe rust severity T1 (%). Adult stripe rust T1 scores refer to score phenotypes at stem elongation (Zadoks 30) of the wheat growth development, and for adult disease data without time point qualifiers refers to the score phenotypes from heading to flowering (Zadoks 55 to 65). The experiments were carried out over 2 years from 2012 to 2013.

Lines were genotyped with TCAP-90K-SWW soft winter wheat chip markers (Table 1). Information about the construction and elaboration of the 90K chip can be found in (http://www.triticeaecap.org/about/project-summary-year-3-2/). This dataset comprised 2075 lines and after deleting markers with more than 50% missing data, minor allele frequency (MAF) less than 5% and removing lines with missing data more than 10%, we retained 4670 markers and 1693 lines. We selected 100, 300, 600 and 1000 wheat genotypes as the TRS size in this dataset.

#### Wheat dataset 2

Phenotypic data for seven traits were analyzed in this dataset 2: lodging (%), plant height (cm), test weight (pounds/bu), grain yield (kg/ha), grain protein (%), lodging2 (%) and heading date (Julian days). Two different scores of lodging were estimated on the field in different developmental stage of the crop (before and after anthesis). The experiments were carried out in Aberdeen over one year (2011) in three experimental trials under three treatments: low nitrogen in dry condition, normal nitrogen in dry condition and normal nitrogen in irrigated condition. Lines were also genotyped with TCAP-90K-SWW soft winter wheat chip markers. This dataset 2 comprised of 537 genotypes and after the same filtering process performed on dataset 1, we retain 5074 markers and 528 lines. In this dataset, 100, 200, 300 and 400 wheat genotypes were selected as the TRS.

### Methods

#### Cross validation experiment

The experimental setup of this study is illustrated in Fig. 3. We started each replication of the experiment by selecting 50 individuals at random, which will create the TS. From the remaining candidates, a specified number of individuals were selected to create the TRS using the STPGA package. In this study, we used two methods for different optimization procedures. When the method does not use the genetic information from the TS to construct the TRS, we called this method as *U-Opt*, or when the TS genetic information is used to build the TRS, we named as *T-Opt* (Fig. 1). For each of these methods, four different optimization criteria were used for optimization of the TRS (A-opt, D-opt, CD_*mean*_ and PEV_*mean*_), which are described above. Once a TRS is identified, a GBLUP model was fitted using the phenotypic and genotypic data on the TRS and the accuracies of the models were evaluated by calculating Pearson correlations between the predicted values and the phenotype values in the TS. The same TS was also estimated using TRS constructed by random sampling. Random sampling was replicated 100 times and the average accuracy was calculated. We have replicated this experiment 30 times. Replicated estimation of the accuracies on the left out TS sample (50 randomly selected genotypes) gives an indication of the generalization performance of genomic prediction models, i.e., the distribution of the prediction performance on an unobserved TS and the mean of this distribution has the same expectation for any TS size (expected accuracy of the model) with sampling variance inversely proportional to the TS size.

#### Optimal designs for genomic prediction

The quality of the inferences and predictions from a genomic prediction model depends on the quality of the experimental data that is used to train these models. Optimal design of experiments deals with planning of experiments so that the available resources are used optimally for the sake of the inferences needed by the researcher^42,43,46,59–64^.

In this paper, we deal with the exact optimal design problem of selecting a set of *n*_*TRS*_ genotypes from a set of *n*_*C*_ candidate genotypes (${n}_{C} > {n}_{TRS}$) in the context of genomic prediction, i.e., in the context of the study of relationships of the type $y=g(x,\theta )$, where a trait of interest *y*, is thought of as a real-valued parametric model function of genome-wide markers $x=({x}_{1},{x}_{2},\ldots ,{x}_{p})$. The parameter value *θ*, is assumed to lie in a parameter domain ${\rm{\Theta }}$. The purpose of the researcher is to use experimental data to make inferences about a function of *θ*; for example, a linear combination of the parameters, or genotypic values for the trait for a set of genotypes.

The first component of this design optimization problem is an objective function, i.e., a design criterion. The second component is a method to look for solutions that optimize the design criterion. The choice of a design criterion will usually depend on the functional form of the regression model $g(x,\theta )$ assumed.

Ridge regression based criteria: If a linear relationship between the response and independent variables is assumed, for a set of individuals that are selected in the training set, we can write the resulting model that describes the relationship between the trait values and the genomic features as

$${y}_{TRS}={X}_{TRS}\beta +\varepsilon ,$$

where *X*_*TRS*_ is the ${n}_{TRS}\times p$ design matrix for genomic features in the training set, ${\beta }_{p\times 1}$ is the vector of regression parameters (effects of genomic features), ${y}_{{n}_{TRS}\times 1}$ is the vector of trait values and ${\varepsilon }_{{n}_{TRS}\times 1}=({\varepsilon }_{1},{\varepsilon }_{2},\ldots ,{\varepsilon }_{{n}_{TRS}})^{\prime}$ is the vector of residual terms. With ${I}_{{n}_{TRS}}$ as the ${n}_{TRS}\times {n}_{TRS}$ identity matrix, the model is represented by the expectation vector and covariance matrix of ${y}_{TRS}$,

$$E({y}_{TRS})={X}_{TRS}\beta ,cov({y}_{TRS})={\sigma }^{2}{I}_{{n}_{TRS}}.$$

If we also assume normality, we can write ${y}_{TRS}\sim {N}_{n}({X}_{TRS}\beta ,{\sigma }^{2}{I}_{{n}_{TRS}})$. When selecting individuals to the TRS, we may wish to estimate a set linear combination of the regression coefficients defined by $\gamma =C\beta$ for a $l\times p$ matrix *C* as precisely as possible by maximizing the information in the sample about the parameter *γ*. For example, for predicting responses in the test set ${\hat{y}}_{TS}={X}_{TS}\hat{\beta }$, we may want to choose a design so as to maximize the relevant information by minimizing the covariance matrix of ${X}_{TS}\hat{\beta }$, i.e., by minimizing prediction error variance (PEV).

When *X*_*TRS*_ has full column rank, we can use ordinary least squares (OLS) estimators for $\gamma =C\beta$. The formula in OLS estimator of *γ* is given by $\hat{\gamma }=C{({X^{\prime} }_{TRS}{X}_{TRS})}^{-1}{X^{\prime} }_{TRS}{y}_{TRS}$ with the sampling covariance matrix ${\sigma }^{2}C{({X^{\prime} }_{TRS}{X}_{TRS})}^{-1}C^{\prime}$.

When the number of columns of *X*_*TRS*_ is large or there is collinearity among the columns of *X*_*TRS*_, we might want to use ridge regression estimators for *γ* with the formula $$\hat{\gamma }=C{({X^{\prime} }_{TRS}{X}_{TRS}+\lambda {I}_{p})}^{-1}{X^{\prime} }_{TRS}{y}_{TRS}$$ for some choice of *λ* > 0, the covariance matrix of this ridge estimator is given by $${\sigma }^{2}C{({X^{\prime} }_{TRS}{X}_{TRS}+\lambda {I}_{p})}^{-1}$$$${X^{\prime} }_{TRS}{X}_{TRS}{({X^{\prime} }_{TRS}{X}_{TRS}+\lambda {I}_{p})}^{-1}C^{\prime} $$. For small *λ*, an approximate the covariance matrix of ridge estimator is given by

1 $${\sigma }^{2}C{({X^{\prime} }_{TRS}{X}_{TRS}+\lambda {I}_{p})}^{-1}C^{\prime} $$

since the limit $${\mathrm{lim}}_{\lambda \to {0}^{+}}{(A+\lambda I)}^{-1}$$ is a generalized inverse of *A*, and $${\mathrm{lim}}_{\lambda \to {0}^{+}}\,{(A+\lambda I)}^{-1}A\,{\mathrm{lim}}_{\lambda \to {0}^{+}}\,{(A+\lambda I)}^{-1}=$$$${\mathrm{lim}}_{\lambda \to {0}^{+}}{(A+\lambda I)}^{-1}$$.

Furthermore, prediction error variance for estimating the $C{X}_{TS}\beta$ with ridge regression is approximately equal to

2 $${\sigma }^{2}C{X}_{TS}{({X^{\prime} }_{TRS}{X}_{TRS}+\lambda {I}_{p})}^{-1}{X^{\prime} }_{TS}C^{\prime} .$$

*X*_*TS*_ is assumed to have *n*_*TS*_ rows. We assume that $n={n}_{C}+{n}_{TRS}$ and ${n}_{C}={n}_{TRS}+{n}_{R}$, where *n*_*R*_ is the number of samples not selected into training set.

Splitting the columns of the design matrix *X*_*TRS*_ as ${X}_{TRS}=({X}^{F},{X}^{R})$, where *X*^*F*^ contains the effects modeled without ridge penalty and *X*^*R*^ contains the terms modeled with ridge penalty, the covariance matrix concerning the estimation of shrunk coefficients is approximately equal to

3 $${({X^{\prime} }_{TRS}^{R}Q{X}_{TRS}^{R}+\lambda {I}_{p})}^{-1}$$

with $$Q=I-{X}^{F}{({X^{\prime} }^{F}{X}^{F})}^{-1}{X^{\prime} }^{F}$$.

Based on the formula based on ridge regression, we can define the following design criteria:

1. A-opt:
  4 $$trace[C{({X^{\prime} }_{TRS}{X}_{TRS}+\lambda {I}_{p})}^{-1}C^{\prime} ].$$
2. D-opt:
  5 $$-\,log|C{({X^{\prime} }_{TRS}{X}_{TRS}+\lambda {I}_{p})}^{-1}C^{\prime} |.$$
3. CD_*mean*_:
  6 $$mean[diag(C{X}_{TS}{({X^{\prime} }_{TRS}{X}_{TRS}+\lambda {I}_{p})}^{-1}{X^{\prime} }_{TS}C^{\prime} )/diag(C{X}_{TS}{X^{\prime} }_{TS}C^{\prime} ))],$$
  the division is element-wise.
4. PEV_*mean*_:

7 $$mean[diag(C{X}_{TS}{({X^{\prime} }_{TRS}{X}_{TRS}+\lambda {I}_{p})}^{-1}{X^{\prime} }_{TS}C^{\prime} )].$$

We are looking to minimize either of these criteria with respect to *X*_*TRS*_. For PEV_*mean*_ and CD_*mean*_, if the test is unknown, the design matrix for the remaining set (the genomic feature matrix for the genotypes in the Candidate set not selected in the TRS), *X*_*R*_, replaces *X*_*TS*_.

The contrast matrix *C* is used when the interest is in estimating a linear function of the effects of genomic features or predictions for individuals. The dimensions of contrast matrix *C* is *l* × *p* ($l\le p$) for A-opt and D-opt where *l* is the number of contrasts in genomic features (linear combination of the genomic features in *X*); $l\times {n}_{TS}$ ($l\le {n}_{TS}$) for CD_*mean*_ and PEV_*mean*_, where this time *l* is the number of contrasts in individuals (linear combination of the predictions for individuals in the test set).

Note that calculation of 4, 5, 6, or 7 involve calculation of the inverse or determinant of a *p* × *p* matrix. This makes these formula unpractical because *p*, the number of genomic features, can be very large. An approach that has been recommended is to use dimension reduction techniques before applying ridge regression related criteria such as A-opt, D-opt, CD_*mean*_ and PEV_*mean*_ with a few extracted features from the genome-wide feature matrix to decrease the computational demands^26,65^. For instance, approximations to the A-opt, D-opt, CD_*mean*_ and PEV_*mean*_ given by Equations 4, 5, 6 and 7 can be obtained by using the first few PCs of the genome-wide marker matrix. Let *P* be the matrix of first $k\ll min(p,n)$ (*k* is much smaller than *min*(*p*, *n*)) PCs partitioned as:

$$P=[\frac{{P}_{C}}{{P}_{TS}}],$$

where *P*_*C*_ is the matrix of PCs for the individuals in the candidate set and *P*_*TS*_ is the matrix of PCs for the individuals in the test set. Now, the design criteria can be calculated based on the matrix *P*, instead of the feature matrix *X*. This formulation is computationally efficient since the order of ${P^{\prime} }_{TRS}{P}_{TRS}+\lambda I$ is the number of columns of *P* and, in general, relatively few principal components will contain most of the variation in the feature matrix.

Out of these four criteria, A-opt and D-opt criteria focus on the optimality of the regression coefficient estimates of the models, while the other two criteria focus on the optimality of the predictions from these models. If the aim of modeling is to estimate the effects of each of the predictor variables in *X* as in the case of association studies, we expect that A-opt and D-opt criterion will be preferable to PEV_*mean*_ and CD_*mean*_. In situations where accurate predictions from the models are sought PEV_*mean*_ and CD_*mean*_ are expected to perform better. However, note that accurate estimation of the model parameters usually leads to accurate predictions, so these approaches are complementary. In relation to this, A-opt and D-opt do not use the test genotype information even when it is present; on the other hand, the other two criteria, PEV_*mean*_ and CD_*mean*_, are designed to use the test information when it is available. The D-opt criterion does not involve matrix inversion and is the most computationally efficient method among these.

Relationship to Mixed Model Based Criteria: Many important statistical regression models can be expressed as mixed models and these models are also widely used model in prediction of quantitative traits, and genome-wide association studies. We can show that the ridge regression related criteria are related to previously suggested mixed model based criteria.

A linear mixed-effects model for a *n*-dimensional response variable *y*, *n* × *p* design matrix of fixed effects, $n\times q$ design matrix of random effects is defined as:

$$y=W\beta +Zu+\varepsilon ;$$

where $\varepsilon \sim {N}_{n}(0,R)$ is independent of $u\sim {N}_{q}(0;G),$ *β* is the *p* dimensional fixed effects, *G* is a $q\times q$ covariance matrix and *R* is a $n\times n$ covariance matrix. The assumptions of the linear mixed-effects model imply $E(y|W;Z)=W\beta$, $y\sim {N}_{n}(W\beta ;ZGZ^{\prime} +R)={N}_{n}(W\beta ;V)$ with *V* defined as $ZGZ^{\prime} +R$.

Henderson’s mixed-model equations^66^ can be used to estimate the standard errors of the fixed and random effects. For a given design, the inverse of the coefficient matrix is written as

$${[\begin{array}{cc}W^{\prime} {R}^{-1}W & W^{\prime} {R}^{-1}Z\\ Z^{\prime} {R}^{-1}W & Z^{\prime} {R}^{-1}Z+{G}^{-1}\end{array}]}^{-1}=[\begin{array}{cc}{H}_{11} & {H}_{12}\\ {H^{\prime} }_{12} & {H}_{22}\end{array}]$$

where *H*_11_, *H*_12_, and *H*_22_ are, respectively, $p\times p$, $p\times q$, and $q\times q$ sub-matrices. Using this notation, the sampling covariance matrix for the BLUE (best linear unbiased estimator) of *β* is given by $cov(\beta )={H}_{11}={(W^{\prime} {V}^{-1}W)}^{-1}$ that the sampling covariance matrix of the prediction errors $(\hat{u}-u)$ (We consider $\hat{u}-u$ rather than $\hat{u}$ as the latter includes variance from both the prediction error and the random effects *u* themselves.) is given by

8 $$cov(\hat{u}-u)={H}_{22}=G-GZ^{\prime} \,PZG$$

for $P={V}^{-1}-{V}^{-1}W{(W^{\prime} {V}^{-1}W)}^{-}W^{\prime} {V}^{-1}$ and that the sampling covariance of estimated effects and prediction errors is given by $cov(\beta ,\hat{u}-u)={H}_{12}=-{(W^{\prime} {V}^{-1}W)}^{-1}W^{\prime} {V}^{-1}ZG$. The standard errors of the fixed and random effects are obtained, respectively, as the square roots of the diagonal elements of *H*_11_ and *H*_22_. In addition, using the above definitions, $cov(u|y)=G-GZ^{\prime} {V}^{-1}ZG={(Z^{\prime} {R}^{-1}Z+{G}^{-1})}^{-1}$.

The variance-covariance matrix of $C^{\prime} (\hat{u}-u)$ given by $C^{\prime} {H}_{22}C$. This is named the prediction error variance whose trace is minimized for selection of training populations. A more recent design criterion is the generalized coefficient of determination (CD)^37,40,67^ for the random terms ${c^{\prime} }_{i}(\hat{u}-u)$, $i=1,\ldots ,l$:

$$\sum _{i=1}^{l}\,\frac{{c^{\prime} }_{i}G{c}_{i}-{c^{\prime} }_{i}{H}_{22}{c}_{i}}{{c^{\prime} }_{i}G{c}_{i}}$$

for a set of contrasts *c*_*i*_. Training populations can be selected to maximize CD or equivalently to minimize

$$\sum _{i=1}^{l}\,\frac{{c^{\prime} }_{i}{H}_{22}{c}_{i}}{{c^{\prime} }_{i}G{c}_{i}}.$$

In a mixed model, genetic information in the form of a pedigree or marker allele frequencies can be used in the form of an additive genetic similarity matrix that describes the similarity based on additive genetic effects (GBLUP). For the ${n}_{TRS}\times 1$ response vector ${y}_{TRS}$, the GBLUP model can be expressed as

9 $${y}_{TRS}={W}_{TRS}\beta +{Z}_{TRS}g+e$$

where ${W}_{TRS}$ is the $n\times q$ design matrix for the fixed effects, *β* is a $q\times 1$ vector of fixed effect coefficients, *Z*_*TRS*_ is the $n\times \ell$ design matrix for the $\ell$ dimensional random effects; the vector random effects $(g^{\prime} ,e^{\prime} )^{\prime}$ is assumed to follow a multivariate normal (MVN) distribution with mean 0 and covariance

10 $$(\begin{array}{cc}{\sigma }_{g}^{2}A & 0\\ 0 & {\sigma }_{e}^{2}{I}_{{n}_{TRS}}\end{array})$$

where *A* is the $\ell \times \ell$ additive genetic similarity matrix. For the GBLUP model, the formula for prediction error variance becomes:

11 $$C{(Z^{\prime} QZ+\lambda {A}^{-1})}^{-1}C^{\prime} $$

and the corresponding formula for coefficient of determination becomes:

12 $$\sum _{i=1}^{l}\,\frac{{c^{\prime} }_{i}(A-\lambda {(Z^{\prime} QZ+\lambda {A}^{-1})}^{-1}){c}_{i}}{{c^{\prime} }_{i}A{c}_{i}},$$

where $$\lambda ={\sigma }_{\varepsilon }^{2}/{\sigma }_{g}^{2}$$.

The GBLUP model is equivalent to a mixed model in which the additive marker effects are estimated via rrBLUP model^68^.

13 $${y}_{TRS}={W}_{TRS}\beta +{X}_{TRS}b+e$$

where ${W}_{TRS}$ is the ${n}_{TRS}\times q$ design matrix for the fixed effects, *β* is a $q\times 1$ vector of fixed effect coefficients, ${X}_{TRS}$ is the ${n}_{TRS}\times p$ design matrix for the genomic features and *b* is the *p* dimensional random effects which represent the additive effects of the genomic features; $(b^{\prime} ,e^{\prime} )^{\prime}$ follows a MVN distribution with mean 0 and covariance

$$(\begin{array}{cc}{\sigma }_{b}^{2}{I}_{p} & 0\\ 0 & {\sigma }_{e}^{2}{I}_{{n}_{TRS}}\end{array}).$$

The formula for the prediction error variance and the coefficient of determination for predicting $C{X}_{TS}b$ for the rrBLUP model are given by

14 $$C{X}_{TS}{({X^{\prime} }_{TRS}Q{X}_{TRS}+\lambda {I}_{p})}^{-1}{X^{\prime} }_{TS}C^{\prime} $$

and

$$\sum _{i=1}^{l}\,(1-\frac{\lambda {c^{\prime} }_{i}{X}_{TS}{({X^{\prime} }_{TRS}Q{X}_{TRS}+\lambda {I}_{p})}^{-1}{X^{\prime} }_{TS}{c}_{i}}{{c^{\prime} }_{i}{X}_{TS}{X^{\prime} }_{TS}{c}_{i}})$$

with $\lambda ={\sigma }_{e}^{2}/{\sigma }_{b}^{2}$ and $Q={I}_{{n}_{TRS}}-{W}_{TRS}{({W^{\prime} }_{TRS}{W}_{TRS})}^{-1}{W^{\prime} }_{TRS}$ is a projection matrix orthogonal to the vector subspace spanned by the columns of ${W}_{TRS}$, so that $Q{W}_{TRS}=0$.

Noting that the expressions in 3 and 14 are essentially the same, we can say that the ridge regression based formulation of CD_*mean*_ and PEV_*mean*_ with PCs that account for most of the variation in the genomic features can be used as approximations to GBLUP or rrBLUP based formulations. Computationally, principal components based formulation of the problem is more efficient than the GBLUP based formulation since $\ell$, the dimension of the matrix that needs to be inverted for the GBLUP based formulations ($\ell$ = number of genotypes in the additive relationship matrix *A*) will be larger than the corresponding dimension (number of principal components, $k\ll min(p,\ell )$) in principal components based ridge regression formulations.

### Optimization using STPGA

In our illustrations, we have used the **R** (R Core Team 2018) package STPGA which contains a special genetic algorithm supplemented with a tabu memory property (that keeps track of previously tried solutions and their fitness for a number of iterations), and with a regression of the fitness of the solutions on their coding that is used to form the ideal estimated solution (look ahead property) to search for solutions of generic optimal subset selection problems and is supplemented with predefined design criteria and the functionality for accepting user defined selection criterion. In addition to optimization of A-opt, D-opt, CD_*mean*_ and PEV_*mean*_; the programs can be used with other available design criteria and with user defined criteria. STPGA is available from the Comprehensive R Archive Network (CRAN) at http://CRAN.R-project.org/package=STPGA, and some of the underlying motivations, methodology and results were presented in^26,27,54,55^.

We note that the genetic algorithm is a global optimization approach and the solutions obtained by any run of a global optimization algorithm may be sub-optimal and different solutions can be obtained given different starting populations. Another layer of safety is obtained if the algorithm is started from multiple initial populations and an island model of evolution is used where separate populations are evolved independently for several steps and then the best solutions from these algorithms become the initial solutions to the evolutionary algorithm.

STPGA can be used to find optimized samples based on the full marker matrix or the genomic relationship matrices (Equations 4, 5, 6 and 7 for ridge regression with markers or Equations 11 and 12 for genomic relationships) and their approximations that are obtained using the principal components (replacing the original features *X* used in A-opt, D-opt, CD_*mean*_ and PEV_*mean*_ in Equations 4, 5, 6 and 7 with the few principal components of *X*).

Numerous other algorithms have been proposed for the optimal subset selection problem, many of them are heuristic exchange type algorithms^27,37,44,69,70^. In exchange type algorithms, new solutions are obtained by adding one point and removing another at a time (some exchange algorithms might allow the exchange of more than one design point at once), these algorithms are greedy and are only proven to find the best subset for certain type of design criteria. In general, exchange algorithms are prone to get stuck in local optima. Branch and bound (BB)^71^ is a global exhaustive search method that has proven to be reasonably efficient on practical problems. BB searches the design region by iteratively dividing design region and searching each piece for an optimal solution. BB is often more efficient than straight enumeration because it can eliminate regions that probably do not contain an optimal solution^72^ uses a BB algorithm to find globally best *D*-optimal design for a given design criterion and a set of candidate points. Another method that has been applied to the subset selection problem is simulated annealing^73^. Branch and bound and simulated annealing algorithms require appreciable computation time even for moderately sized problems. The main advantage of the GA in STPGA is that it benefits from parallelism and it is a general purpose optimal subset selection algorithm which can be easily adapted to work with any design criteria. For standard design criteria such as D-opt, A-opt and PEV_*mean*_ other efficient specific purpose algorithms might be preferred (for example, DETMAX algorithm by Mitchell^69^ or Algorithm by Fedorov^44^).

## Supplementary information

Design of training populations for selective 1 phenotyping in genomic prediction 2 Supplementary Materials

## References

[1] Meuwissen T, Hayes B, Goddard M (2001). Prediction of total genetic value using genome-wide dense marker maps. Genetics.

[2] Vazquez A (2012). A comprehensive genetic approach for improving prediction of skin cancer risk in humans. Genetics.

[3] de los Campos G, Hickey J, Pong-Wong R, Daetwyler H, Calus M (2013). Whole-genome regression and prediction methods applied to plant and animal breeding. Genetics.

[4] Wray NR (2013). Pitfalls of predicting complex traits from snps. Nature Reviews Genetics.

[5] Burke W, Psaty B (2007). Personalized medicine in the era of genomics. Jama.

[6] Bielinski, S. *et al*. Preemptive genotyping for personalized medicine: design of the right drug, right dose, right time—using genomic data to individualize treatment protocol. In *Mayo Clinic Proceedings*, vol. 89(1), 25–33 (Elsevier, 2014). 10.1016/j.mayocp.2013.10.021 PMC3932754 24388019

[7] Bernardo R, Yu J (2007). Prospects for genomewide selection for quantitative traits in maize. Crop Science.

[8] Heffner E, Sorrells M, Jannink J (2009). Genomic selection for crop improvement. Crop Science.

[9] Falconer D, Mackay T (1996). Introduction to quantitative genetics.

[10] Isidro, J., Akdemir, D. & Burke, J. Genomic selection. In William, A., Alain, B. & Maarten, V. G. (eds) *The world wheat book*: *a history of wheat breeding*, vol. 3, chap. 32, 1001–1023 (Lavoisier, Paris, 2016).

[11] Habier D, Fernando R, Dekkers J (2007). The impact of genetic relationship information on genome-assisted breeding values. Genetics.

[12] Goddard M (2009). Genomic selection: prediction of accuracy and maximisation of long term response. Genetics.

[13] McClellan J, Susser E, King M (2007). Schizophrenia: a common disease caused by multiple rare alleles. The British Journal of Psychiatry.

[14] Jannink, J.-L., Lorenz, A. J. & Iwata, H. Genomic selection in plant breeding: from theory to practice. *Briefings in functional genomics* elq001 (2010). 10.1093/bfgp/elq001 20156985

[15] Burstin J (2015). Genetic diversity and trait genomic prediction in a pea diversity panel. BMC genomics.

[16] Heslot N, Yang H, Sorrells M, Jannink J (2012). Genomic selection in plant breeding: a comparison of models. Crop Science.

[17] Hayes B, Bowman P, Chamberlain A, Goddard M (2009). Invited review: Genomic selection in dairy cattle: Progress and challenges. Journal of Dairy Science.

[18] Yang J (2010). Common snps explain a large proportion of the heritability for human height. Nature Genetics.

[19] Combs, E. & Bernardo, R. Accuracy of genomewide selection for different traits with constant population size, heritability, and number of markers. *The Plant Genome* **6** (2013).

[20] Saatchi, M., Miraei-Ashtiani, S., Javaremi, A. N. & Mehrabani-Yeghaneh, H. The impact of information quantity and strength of relationship between training set and validation set on accuracy of genomic estimated breeding values. *African Journal of Biotechnology* **9** (2010).

[21] Habier D, Tetens J, Seefried F-R, Lichtner P, Thaller G (2010). The impact of genetic relationship information on genomic breeding values in german holstein cattle. Genetics Selection Evolution.

[22] Clark SA, Hickey JM, Van der Werf JH (2011). Different models of genetic variation and their effect on genomic evaluation. Genet Sel Evol.

[23] Albrecht T (2011). Genome-based prediction of testcross values in maize. Theoretical and Applied Genetics.

[24] Clark SA, Hickey JM, Daetwyler HD, van der Werf JH (2012). The importance of information on relatives for the prediction of genomic breeding values and the implications for the makeup of reference data sets in livestock breeding schemes. Genet Sel Evol.

[25] Pszczola M, Strabel T, Mulder H, Calus M (2012). Reliability of direct genomic values for animals with different relationships within and to the reference population. Journal of dairy science.

[26] Akdemir D, Sanchez JI, Jannink J-L (2015). Optimization of genomic selection training populations with a genetic algorithm. Genet Sel Evol.

[27] Isidro J (2015). Training set optimization under population structure in genomic selection. Theoretical and Applied Genetics.

[28] Lado B (2013). Increased genomic prediction accuracy in wheat breeding through spatial adjustment of field trial data. G3: Genes Genomes Genetics.

[29] Crossa J (2010). Prediction of genetic values of quantitative traits in plant breeding using pedigree and molecular markers. Genetics.

[30] Riedelsheimer C (2012). Genomic and metabolic prediction of complex heterotic traits in hybrid maize. Nature genetics.

[31] Heslot N, Jannink J, Sorrells M (2013). Using genomic prediction to characterize environments and optimize prediction accuracy in applied breeding data. Crop Science.

[32] Lorenz AJ, Smith KP (2015). Adding genetically distant individuals to training populations reduces genomic prediction accuracy in barley. Crop Science.

[33] Bustos-Korts D, Malosetti M, Chapman S, Biddulph B, van Eeuwijk F (2016). Improvement of predictive ability by uniform coverage of the target genetic space. G3: Genes Genomes Genetics.

[34] Whittaker JC, Thompson R, Denham MC (2000). Marker-assisted selection using ridge regression. Genetics Research.

[35] Jacobson A, Lian L, Zhong S, Bernardo R (2014). General combining ability model for genomewide selection in a biparental cross. Crop Science.

[36] Fristche-Neto R, Akdemir D, Jannink J-L (2018). Accuracy of genomic selection to predict maize single-crosses obtained through different mating designs. Theoretical and Applied Genetics.

[37] Rincent R (2012). Maximizing the reliability of genomic selection by optimizing the calibration set of reference individuals: comparison of methods in two diverse groups of maize inbreds (zea mays l.). Genetics.

[38] Jarquin D, Specht J, Lorenz A (2016). Prospects of genomic prediction in the usda soybean germplasm collection: Historical data creates robust models for enhancing selection of accessions. G3: Genes, Genomes, Genetics.

[39] Bustos-Korts, D., Malosetti, M., Chapman, S. & van Eeuwijk, F. Modelling of genotype by environment interaction and prediction of complex traits across multiple environments as a synthesis of crop growth modelling, genetics and statistics. In *Crop systems biology*, 55–82 (Springer, 2016).

[40] Laloë D (1993). Precision and information in linear models of genetic evaluation. Genetics Selection Evolution.

[41] Smith K (1918). On the standard deviations of adjusted and interpolated values of an observed polynomial function and its constants and the guidance they give towards a proper choice of the distribution of observations. Biometrika.

[42] Kiefer, J. Optimum experimental designs. *Journal of the Royal Statistical Society*. *Series B* (*Methodological*) 272–319 (1959).

[43] Fisher RA (1960). The design of experiments.

[44] Fedorov, V. V. *Theory of optimal experiments* (Elsevier, 1972).

[45] Silvey, S. *Optimal design*: *an introduction to the theory for parameter estimation*, vol. 1 (Springer Science & Business Media, 2013).

[46] Atkinson, A. & Donev, A. *Optimum experimental designs* (Oxford, 1992).

[47] Pukelsheim F, Rosenberger J (1993). Experimental designs for model discrimination. Journal of the American Statistical Association.

[48] Fedorov, V. V. & Hackl, P. *Model-oriented design of experiments*, vol. 125 (Springer Science & Business Media, 2012).

[49] Akdemir, D. *STPGA*: *Selection of Training Populations by Genetic Algorithm*, https://CRAN.R-project.org/package=STPGA, R package version 4.0 (2017).

[50] Saatchi M (2011). Accuracies of genomic breeding values in american angus beef cattle using k-means clustering for cross-validation. Genetics Selection Evolution.

[51] Wimmer V (2013). Genome-wide prediction of traits with different genetic architecture through efficient variable selection. Genetics.

[52] Hickey J (2014). Evaluation of genomic selection training population designs and genotyping strategies in plant breeding programs using simulation. Crop Science.

[53] Guo Z (2014). The impact of population structure on genomic prediction in stratified populations. Theoretical and applied genetics.

[54] Crossa J (2016). Genomic prediction of gene bank wheat landraces. G3: Genes Genomes Genetics.

[55] Akdemir, D. & Sánchez, J. I. Efficient breeding by genomic mating. *Frontiers in Genetics* **7** (2016). 10.3389/fgene.2016.00210 PMC5126051 27965707

[56] Akdemir, D., Beavis, W., Fritsche-Neto, R., K. Singh, A. & Isidro-Sánchez, J. Multi-objective optimized genomic breeding strategies for sustainable food improvement. *Hered*. 1 (2018). 10.1038/s41437-018-0147-1 PMC6461918 30262841

[57] Poland JA, Rife T (2012). Genotyping-by-sequencing for plant breeding and genetics. The Plant Genome.

[58] Dempster, A., Laird, N. & Rubin, D. Maximum likelihood from incomplete data via the em algorithm. *Journal of the royal statistical society*. *Series B* (*methodological*) 1–38 (1977).

[59] Yates F (1935). Complex experiments. Supplement to the Journal of the Royal Statistical Society.

[60] Fisher, R. A. The arrangement of field experiments. In *Breakthroughs in statistics*, 82–91 (Springer, 1992).

[61] Box, G. E., Hunter, W. G. & Hunter, J. S. *Statistics for experimenters*: *an introduction to design*, *data analysis*, *and model building*, vol. 1 (JSTOR, 1978).

[62] Wynn HP (1984). Jack kiefer’s contributions to experimental design. The Annals of Statistics.

[63] Draper NR, Pukelsheim F (1996). An overview of design of experiments. Statistical Papers.

[64] Pukelsheim, F. *Optimal design of experiments* (SIAM, 2006).

[65] Ruiz, J. S. *Optimal designs in genomic selection*. Ph.D. thesis, (The University of Nebraska-Lincoln, 2015).

[66] Henderson C (1975). Best linear unbiased estimation and prediction under a selection model. Biometrics.

[67] Laloë, D. & Phocas, F. A proposal of criteria of robustness analysis in genetic evaluation. *Livestock Production Science* **80**, 241–256, https://www.sciencedirect.com/science/article/pii/S0301622602000921, 10.1016/S0301-6226(02)00092-1 (2003).

[68] VanRaden PM (2008). Efficient methods to compute genomic predictions. Journal of dairy science.

[69] Mitchell T (1974). An algorithm for the construction of “d-optimal” experimental designs. Technometrics.

[70] Nguyen N, Miller A (1992). A review of some exchange algorithms for constructing discrete d-optimal designs. Computational Statistics & Data Analysis.

[71] Furnival G, Wilson R (1974). Regressions by leaps and bounds. Technometrics.

[72] Welch WJ (1982). Branch-and-bound search for experimental designs based on d optimality and other criteria. Technometrics.

[73] Haines LM (1987). The application of the annealing algorithm to the construction of exact optimal designs for linear–regression models. Technometrics.

