# Supplementary material for: Design of training populations for selective phenotyping in genomic prediction
Source: Sci Rep. 2019 Feb 5;9:1446. doi: 10.1038/s41598-018-38081-6 (PMC6363789; doi:10.1038/s41598-018-38081-6)
Supplement: Supplementary file 1 — Design of training populations for selective 1 phenotyping in genomic prediction 2 Supplementary Materials [file 41598_2018_38081_MOESM1_ESM.pdf]

# *Design of training populations for selective phenotyping in genomic prediction Supplementary Materials*

**Deniz Akdemir**[1*] **and Julio Isidro-Sánchez**[2*]

[1]Cornell University Statistical Consulting Unit, Ithaca, NY, USA.
[2]School of Agriculture and Food Science, University College Dublin, Dublin, Ireland.
[*]da346@cornell.edu; j.isidro@ucd.ie

## ABSTRACT

This file contains the supplementary figures and tables for the main text.

## Supplementary Figures

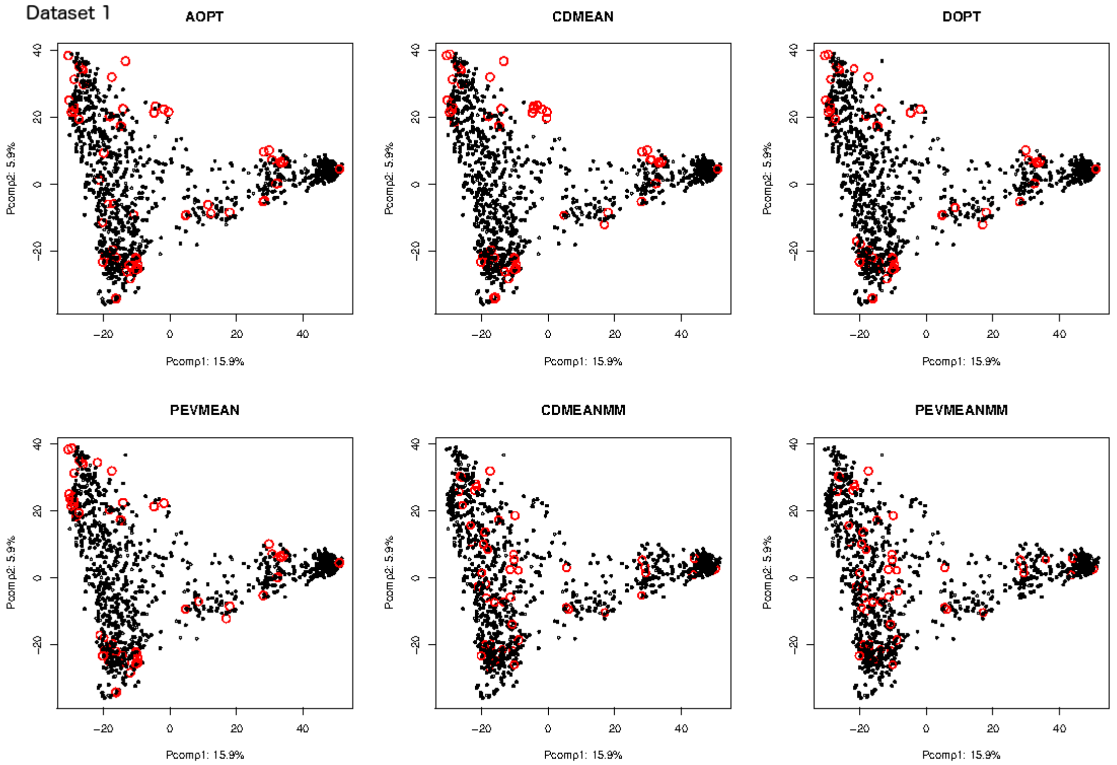


**1.** Figure S1. Genotypes selected from the optimization algorithm are plotted on the principal components 1 and 2 analysis in dataset 1. The genotypes were selected based on AOPT, CDmean, DOPT, PEVmean, CDMEANMM, PEVMEANMM from the STPGA package.

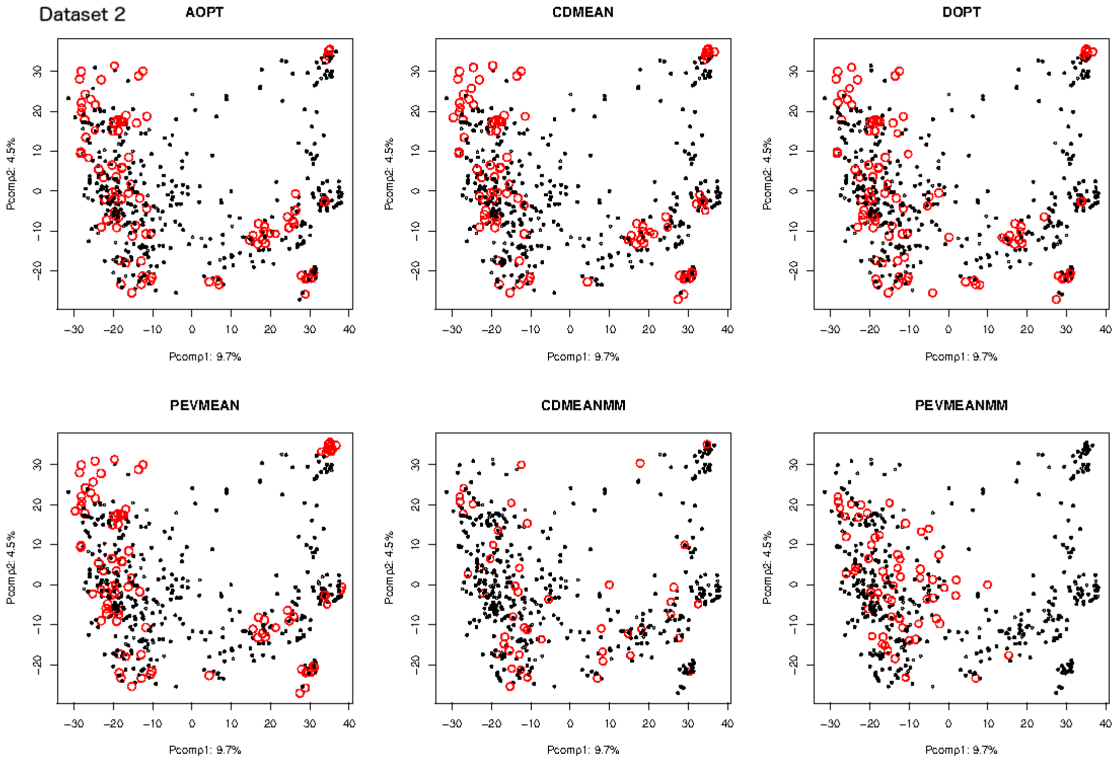


**2.** Figure S2. Genotypes selected from the optimization algorithm are plotted on the principal components 2 and 3 analysis in dataset 2. The genotypes were selected based on AOPT, CDmean, DOPT, PEVmean,CDmeanMM,PEVmeanMM from the STPGA package.

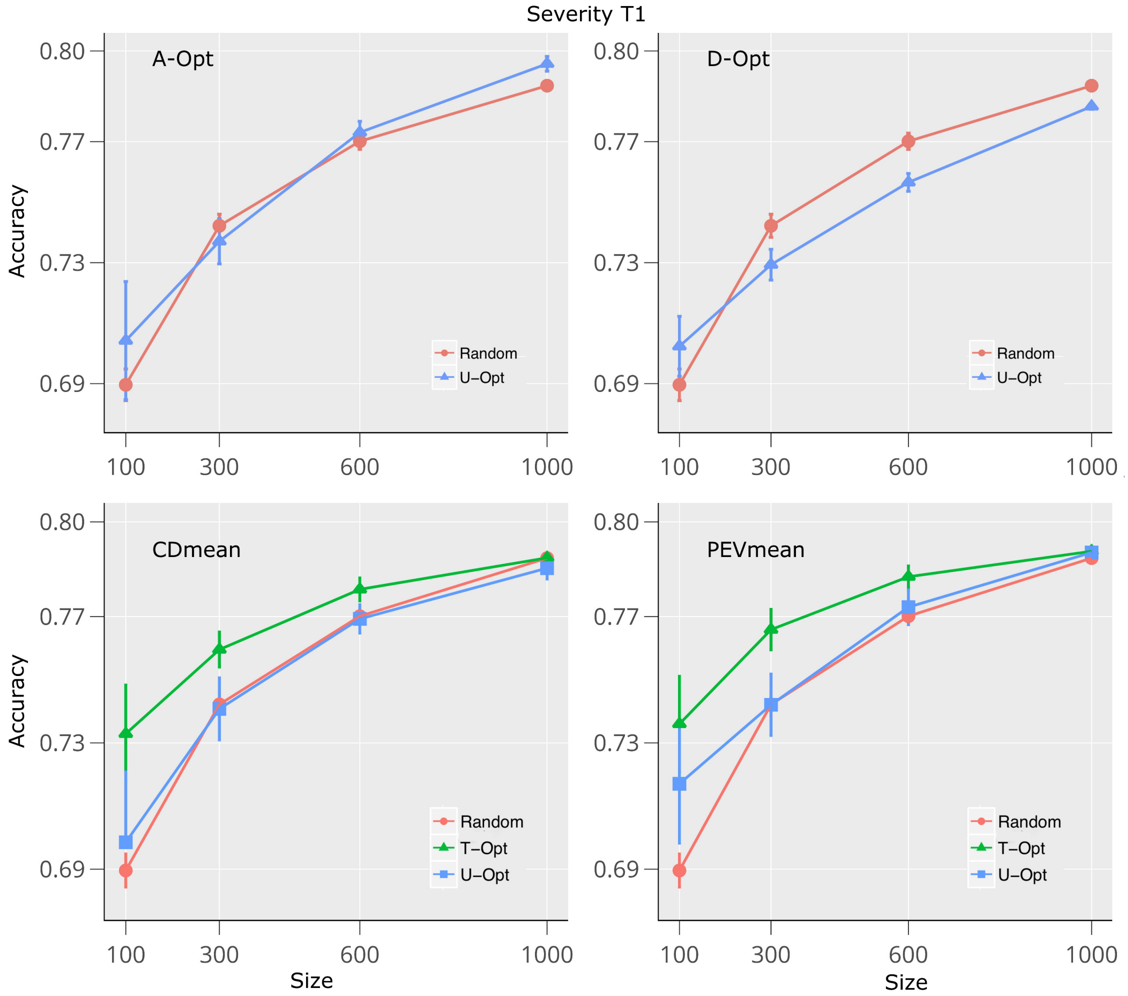


**3.** Figure S3. Prediction accuracies for stripe rust severity time 1 trait using sampling algorithms within STPGA package on dataset 1. Accuracies of the predictions of the test set (TS) genotypes were calculated using 4 different algorithms and 2 methods compared with random sampling. In the U-Opt method, the TS were not used to build the training population set (TRS) while in the T-Opt the optimization algorithm used the TS to build the TRS. The TRS were defined by optimizing A-Opt, D-Opt, CDmean, and PEVmean. Four different population sizes (100, 300, 600 and 1000) were used for the optimization algorithm. Standard error is indicated for each point over 30 (U-Opt and T-Opt) and 100 (random) runs.

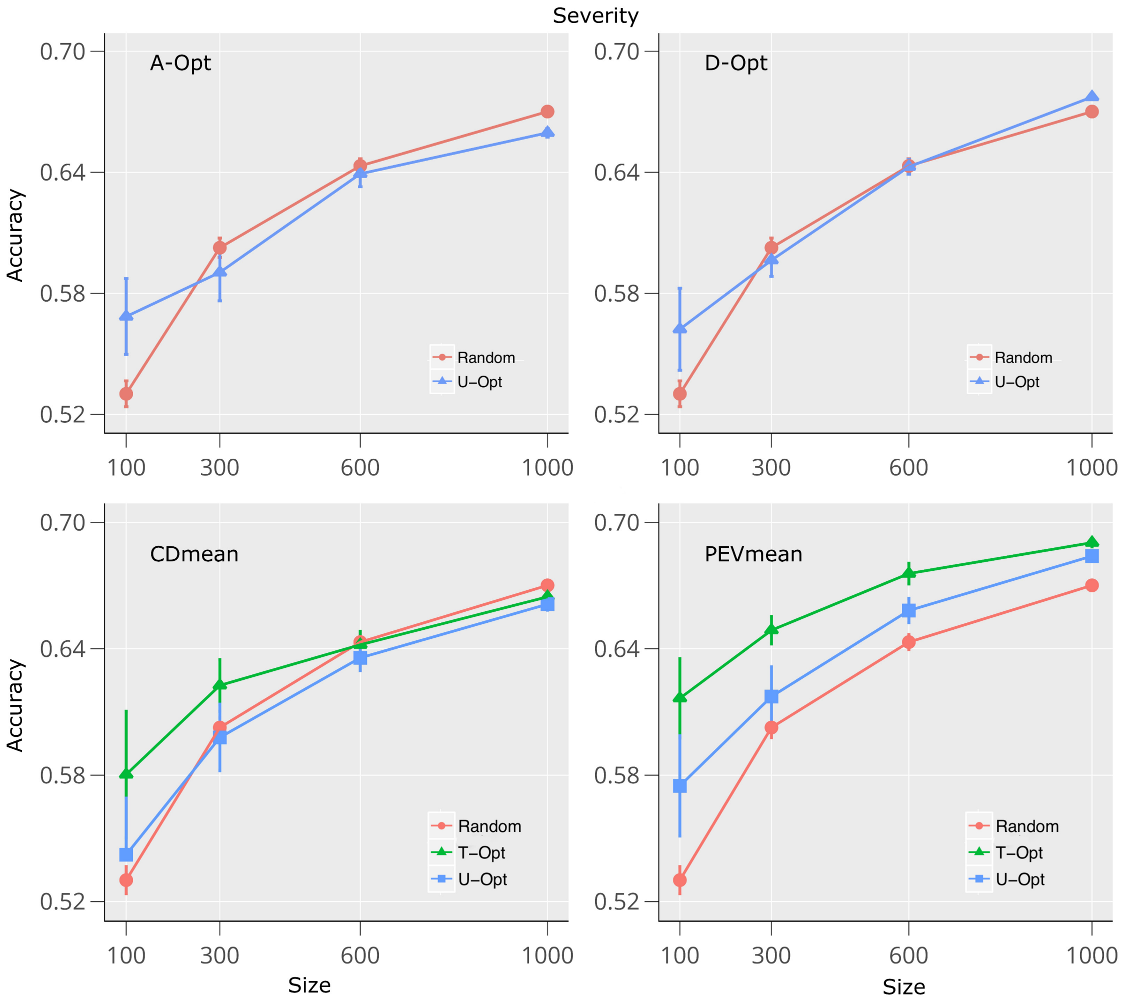


**4.** Figure S4. Prediction accuracies for stripe rust severity percentage trait using sampling algorithms within STPGA package on dataset 1. Accuracies of the predictions of the test set (TS) genotypes were calculated using 4 different algorithms and 2 methods compared with random sampling. In the U-Opt method, the TS were not used to build the training population set (TRS) while in the T-Opt the optimization algorithm used the TS to build the TRS. The TRS were defined by optimizing A-Opt, D-Opt, CDmean, and PEVmean. Four different population sizes (100, 300, 600 and 1000) were used for the optimization algorithm. Standard error is indicated for each point over 30 (U-Opt and T-Opt) and 100 (random) runs.

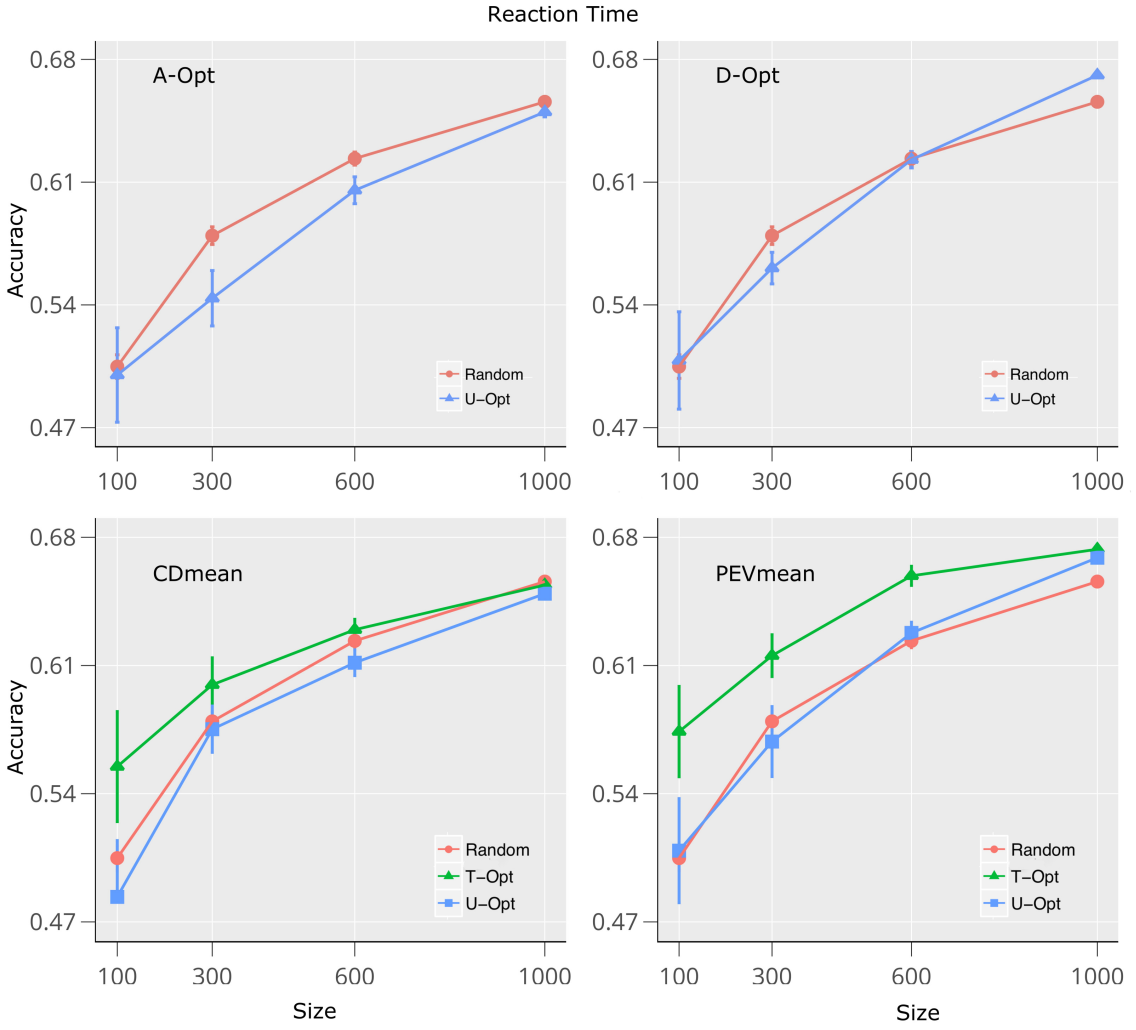


**5.** Figure S5. Prediction accuracies for adult stripe rust Reaction Time 1 trait using sampling algorithms within STPGA package on dataset 1. Accuracies of the predictions of the test set (TS) genotypes were calculated using 4 different algorithms and 2 methods compared with random sampling. In the U-Opt method, the TS were not used to build the training population set (TRS) while in the T-Opt the optimization algorithm used the TS to build the TRS. The TRS were defined by optimizing A-Opt, D-Opt, CDmean, and PEVmean. Four different population sizes (100, 300, 600 and 1000) were used for the optimization algorithm. Standard error is indicated for each point over 30 (U-Opt and T-Opt) and 100 (random) runs.

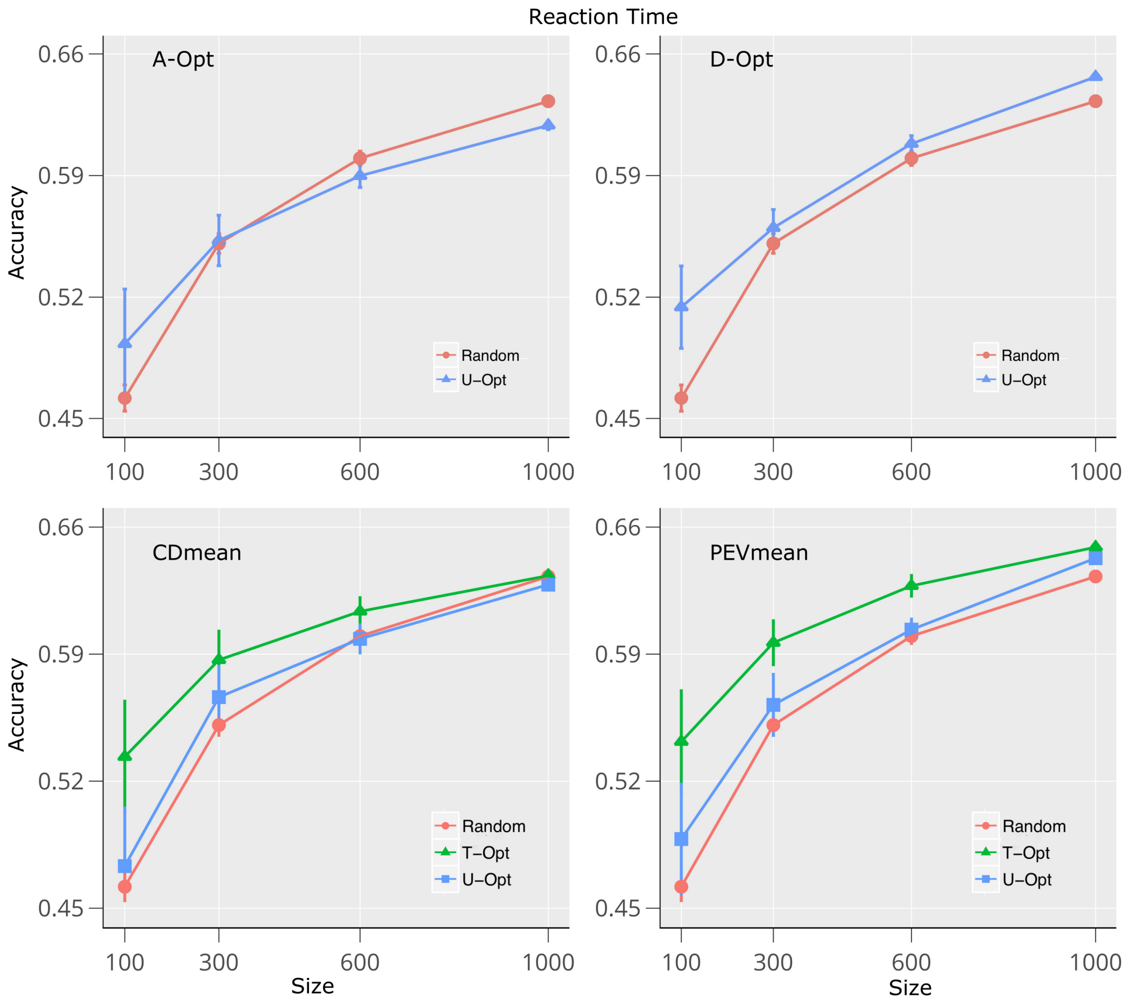


**6.** Figure S6. Prediction accuracies for adult stripe rust Reaction Time percentage trait using sampling algorithms within STPGA package on dataset 1. Accuracies of the predictions of the test set (TS) genotypes were calculated using 4 different algorithms and 2 methods compared with random sampling. In the U-Opt method, the TS were not used to build the training population set (TRS) while in the T-Opt the optimization algorithm used the TS to build the TRS. The TRS were defined by optimizing A-Opt, D-Opt, CDmean, and PEVmean. Four different population sizes (100, 300, 600 and 1000) were used for the optimization algorithm. Standard error is indicated for each point over 30 (U-Opt and T-Opt) and 100 (random) runs.

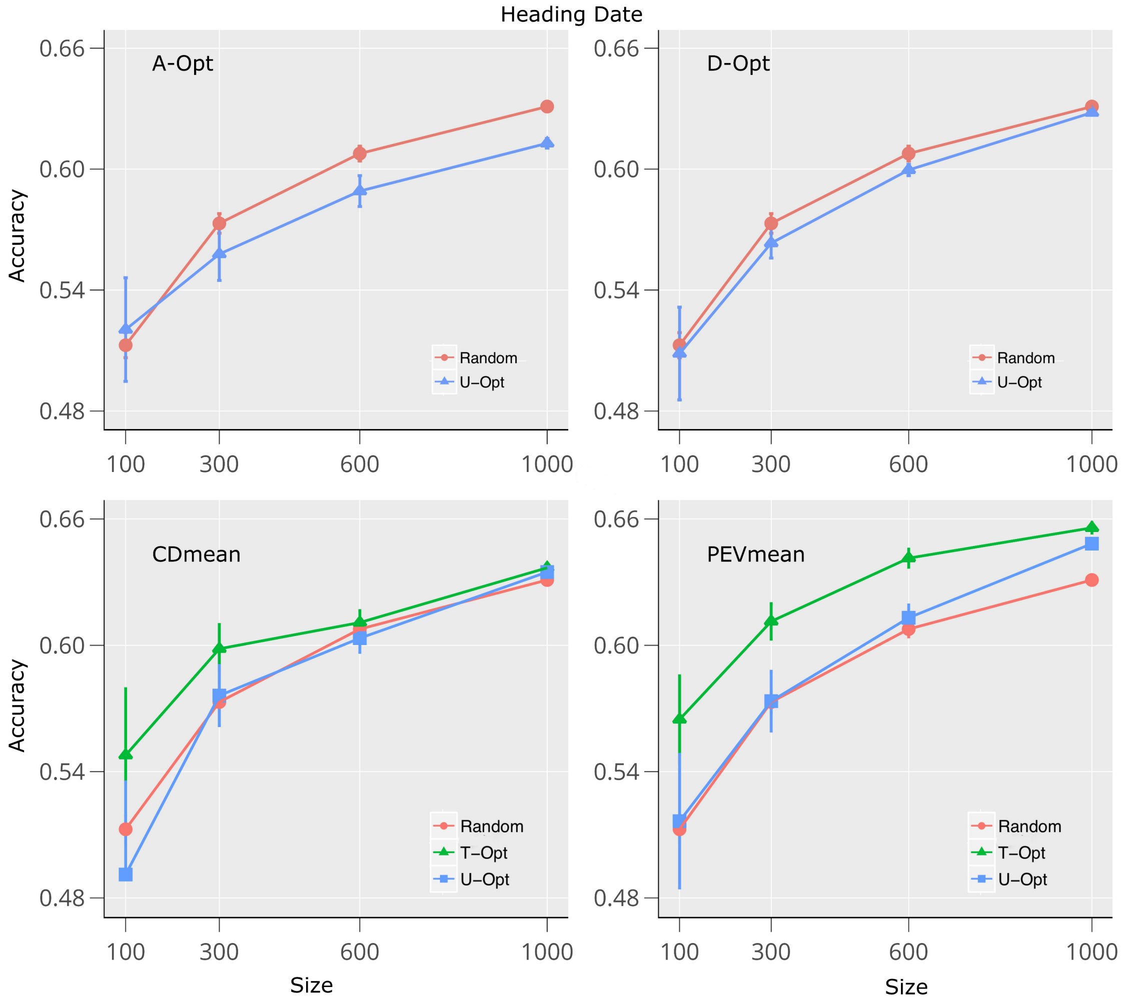


**7.** Figure S7. Prediction accuracies for heading date using sampling algorithms within STPGA package on dataset 1. Accuracies of the predictions of the test set (TS) genotypes were calculated using 4 different algorithms and 2 methods compared with random sampling. In the U-Opt method, the TS were not used to build the training population set (TRS) while in the T-Opt the optimization algorithm used the TS to build the TRS. The TRS were defined by optimizing A-Opt, D-Opt, CDmean, and PEVmean. Four different population sizes (100, 200, 300 and 400) were used for the optimization algorithm. Standard error is indicated for each point over 30 (U-Opt and T-Opt) and 100 (random) runs. (random) runs.

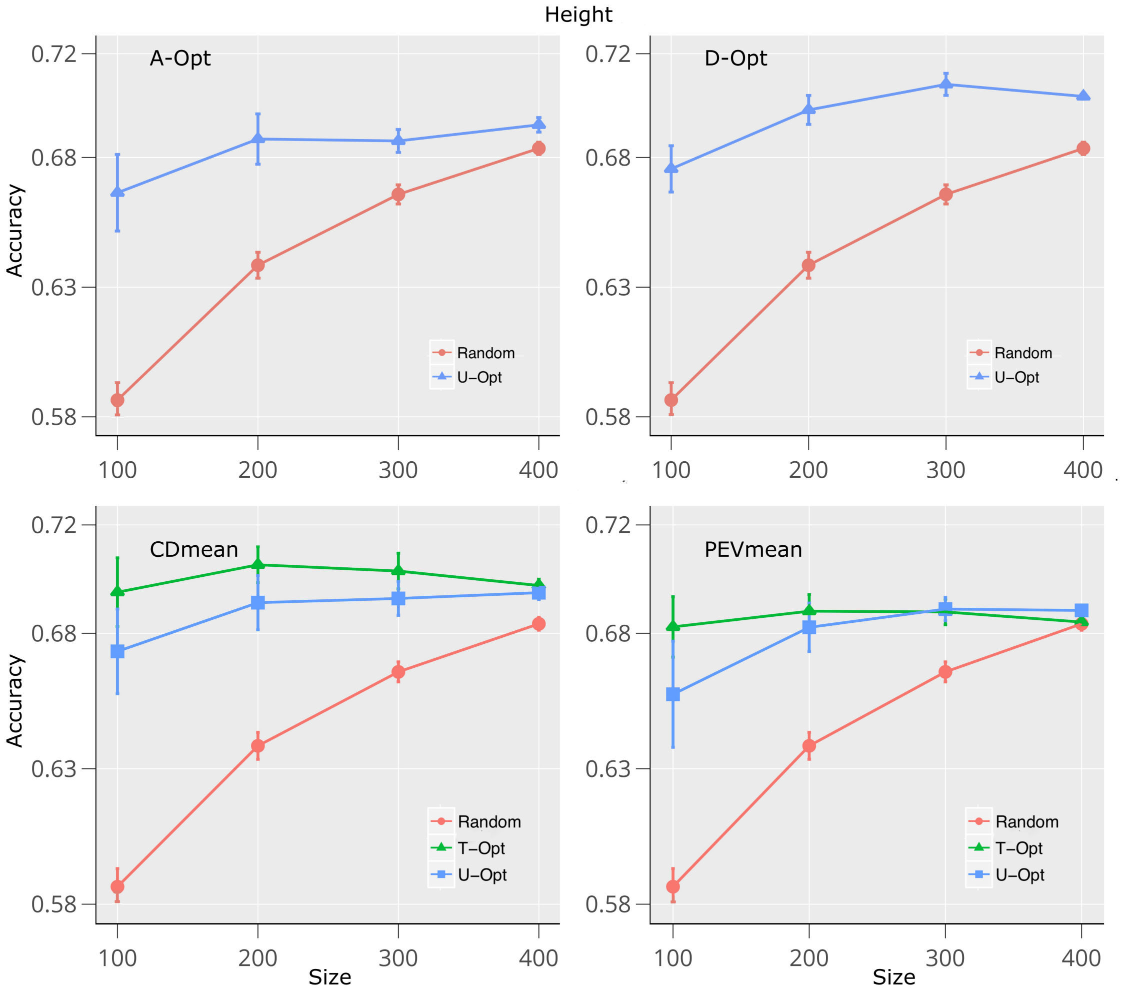


**8.** Figure S8. Prediction accuracies for height using sampling algorithms within STPGA package on dataset 2. Accuracies of the predictions of the test set (TS) genotypes were calculated using 4 different algorithms and 2 methods compared with random sampling. In the U-Opt method, the TS were not used to build the training population set (TRS) while in the T-Opt the optimization algorithm used the TS to build the TRS. The TRS were defined by optimizing A-Opt, D-Opt, CDmean, and PEVmean. Four different population sizes (100, 200, 300 and 400) were used for the optimization algorithm. Standard error is indicated for each point over 30 (U-Opt and T-Opt) and 100 (random) runs. (random) runs.

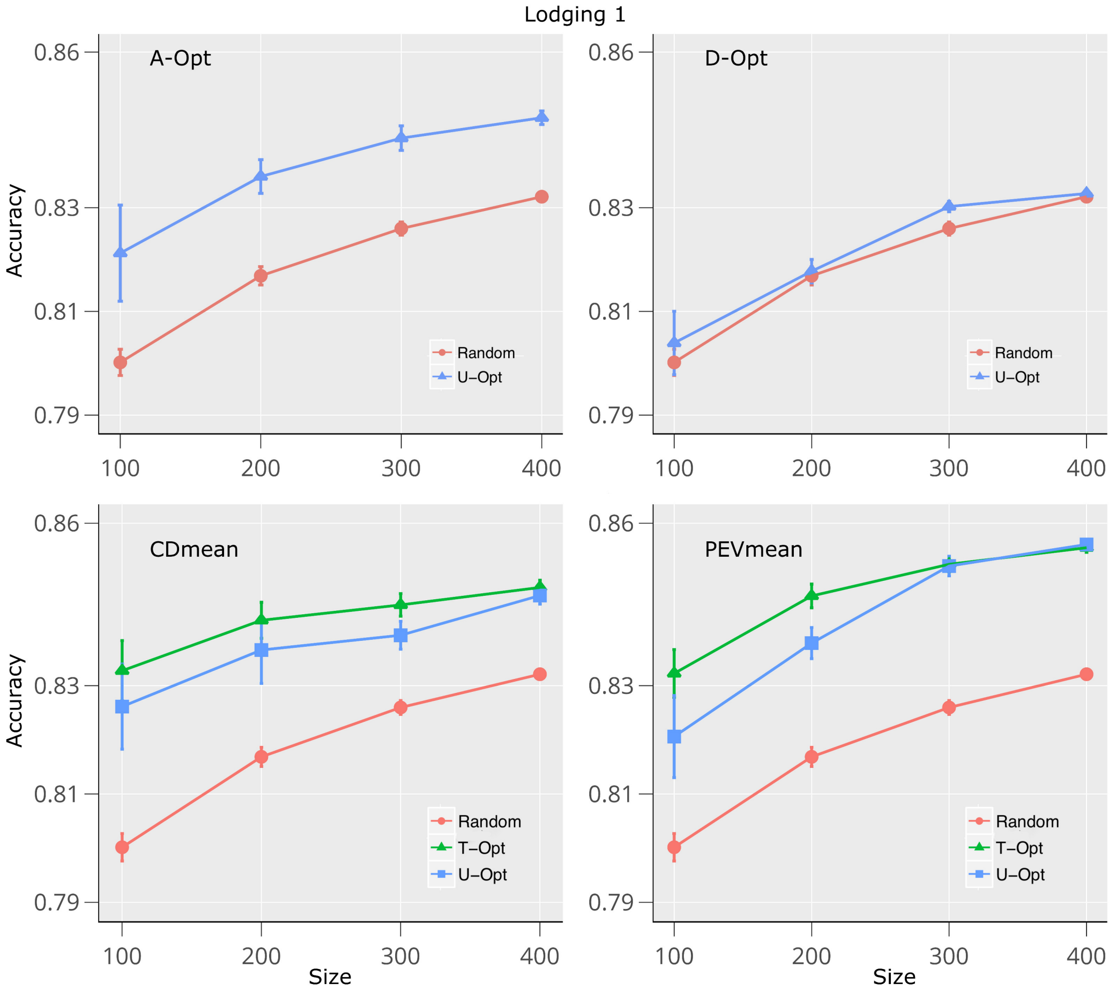


**9.** Figure S9. Prediction accuracies for Lodging time 1 using sampling algorithms within STPGA package on dataset 2. Accuracies of the predictions of the test set (TS) genotypes were calculated using 4 different algorithms and 2 methods compared with random sampling. In the U-Opt method, the TS were not used to build the training population set (TRS) while in the T-Opt the optimization algorithm used the TS to build the TRS. The TRS were defined by optimizing A-Opt, D-Opt, CDmean, and PEVmean. Four different population sizes (100, 200, 300 and 400) were used for the optimization algorithm. Standard error is indicated for each point over 30 (U-Opt and T-Opt) and 100 (random) runs. (random) runs.

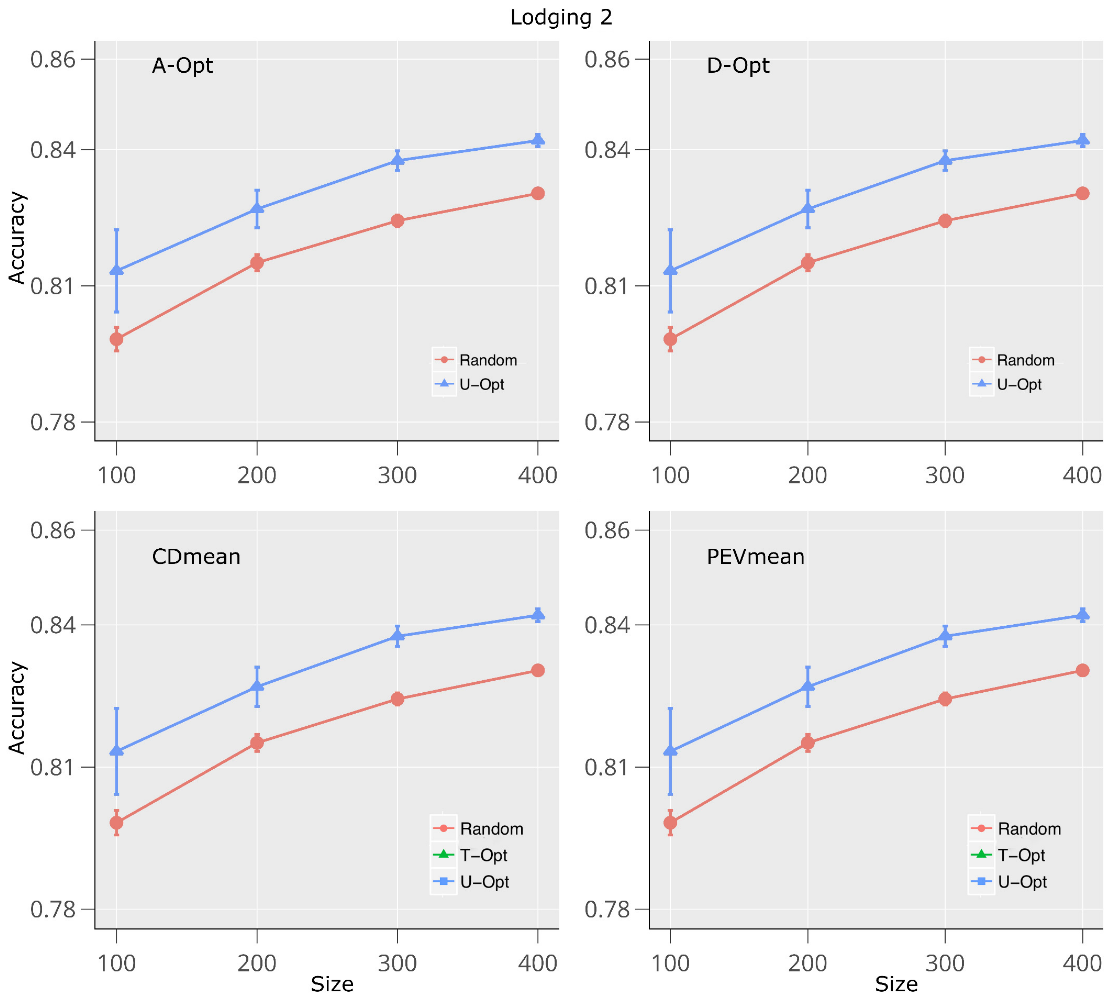


**10.** Figure S10. Prediction accuracies for Lodging time 2 using sampling algorithms within STPGA package on dataset 2. Accuracies of the predictions of the test set (TS) genotypes were calculated using 4 different algorithms and 2 methods compared with random sampling. In the U-Opt method, the TS were not used to build the training population set (TRS) while in the T-Opt the optimization algorithm used the TS to build the TRS. The TRS were defined by optimizing A-Opt, D-Opt, CDmean, and PEVmean. Four different population sizes (100, 200, 300 and 400) were used for the optimization algorithm. Standard error is indicated for each point over 30 (U-Opt and T-Opt) and 100 (random) runs. (random) runs.

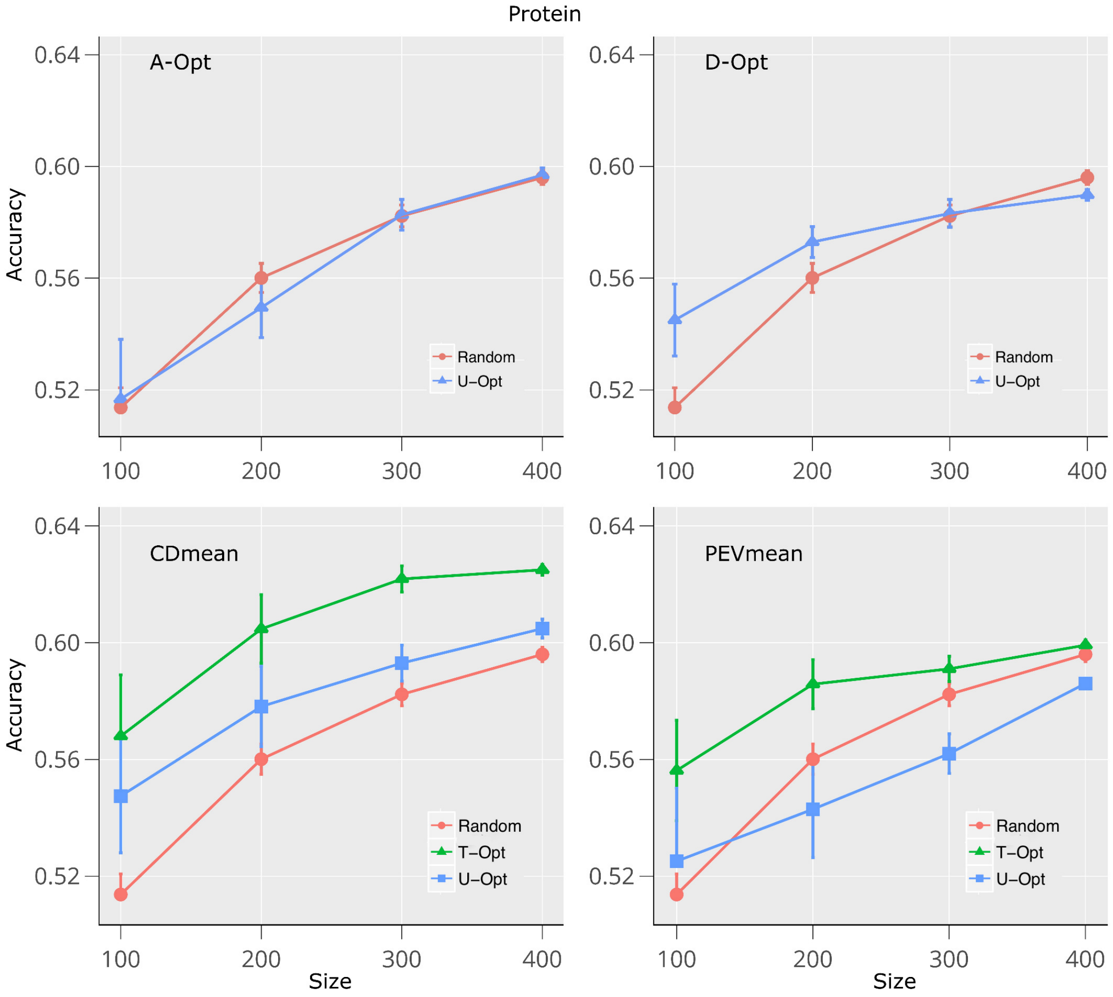


**11.** Figure S11. Prediction accuracies for Protein using sampling algorithms within STPGA package on dataset 2. Accuracies of the predictions of the test set (TS) genotypes were calculated using 4 different algorithms and 2 methods compared with random sampling. In the U-Opt method, the TS were not used to build the training population set (TRS) while in the T-Opt the optimization algorithm used the TS to build the TRS. The TRS were defined by optimizing A-Opt, D-Opt, CDmean, and PEVmean. Four different population sizes (100, 200, 300 and 400) were used for the optimization algorithm. Standard error is indicated for each point over 30 (U-Opt and T-Opt) and 100 (random) runs. (random) runs.

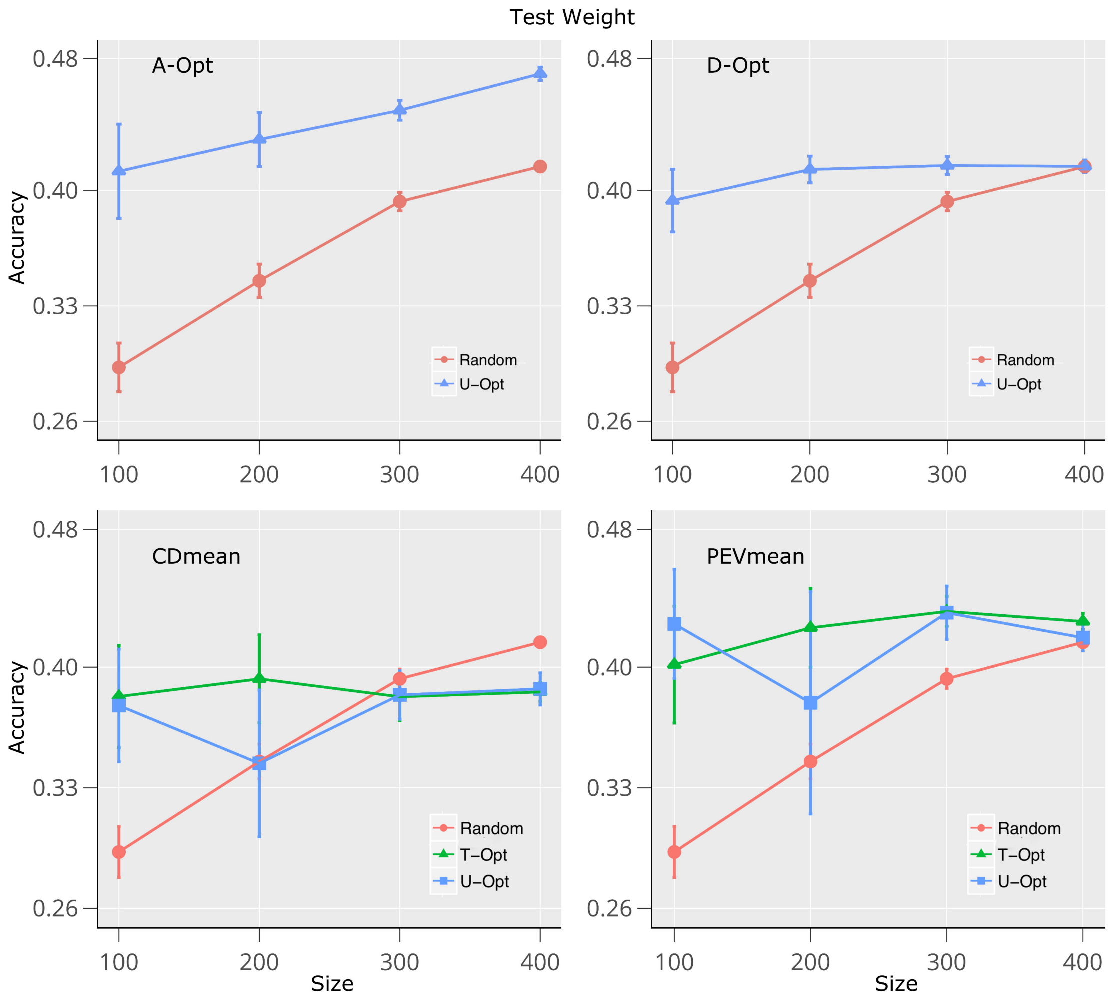


**12.** Figure S12. Prediction accuracies for Test Weight using sampling algorithms within STPGA package on dataset 2. Accuracies of the predictions of the test set (TS) genotypes were calculated using 4 different algorithms and 2 methods compared with random sampling. In the U-Opt method, the TS were not used to build the training population set (TRS) while in the T-Opt the optimization algorithm used the TS to build the TRS. The TRS were defined by optimizing A-Opt, D-Opt, CDmean, and PEVmean. Four different population sizes (100, 200, 300 and 400) were used for the optimization algorithm. Standard error is indicated for each point over 30 (U-Opt and T-Opt) and 100 (random) runs. (random) runs.

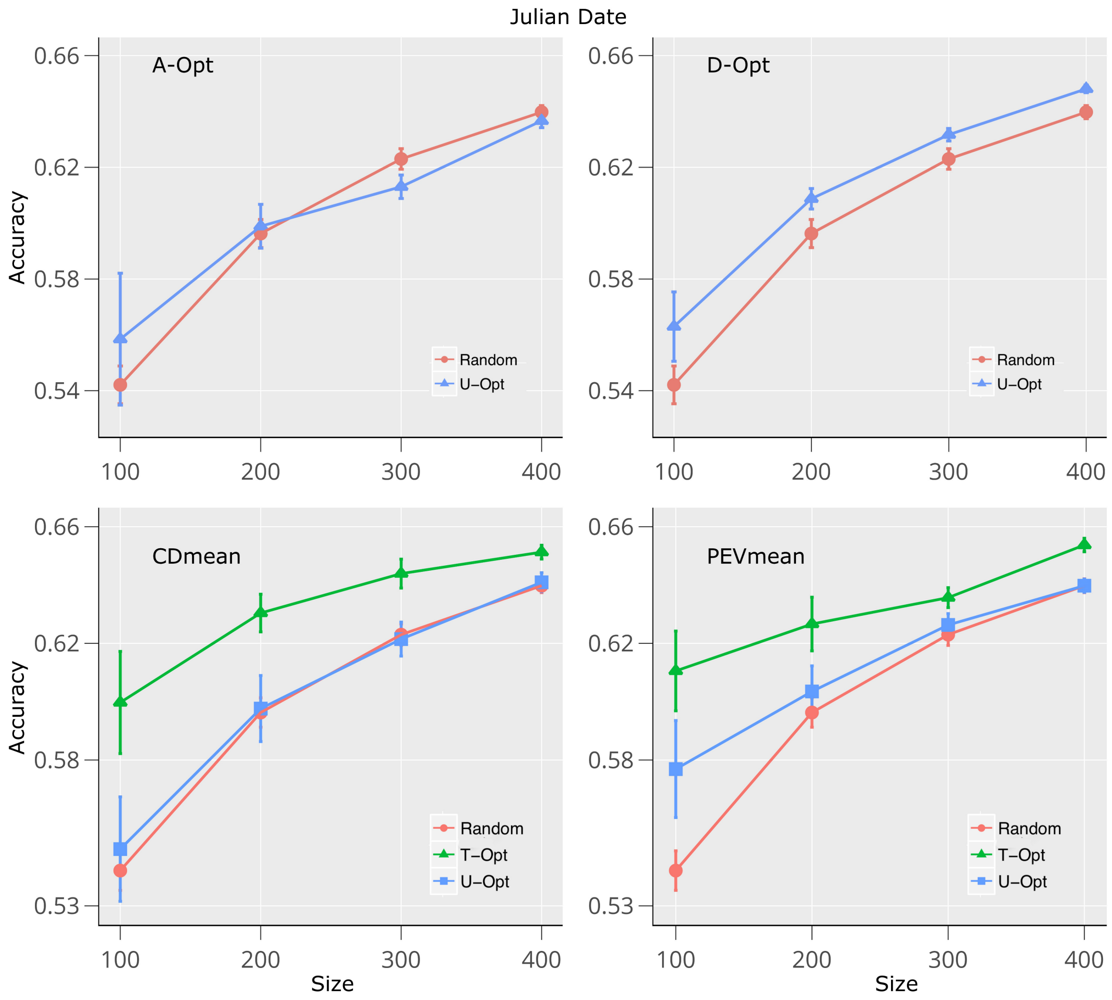


**13.** Figure S13. Prediction accuracies for Test Wight using sampling algorithms within STPGA package on dataset 2. Accuracies of the predictions of the test set (TS) genotypes were calculated using 4 different algorithms and 2 methods compared with random sampling. In the U-Opt method, the TS were not used to build the training population set (TRS) while in the T-Opt the optimization algorithm used the TS to build the TRS. The TRS were defined by optimizing A-Opt, D-Opt, CDmean, and PEVmean. Four different population sizes (100, 200, 300 and 400) were used for the optimization algorithm. Standard error is indicated for each point over 30 (U-Opt and T-Opt) and 100 (random) runs. (random) runs.

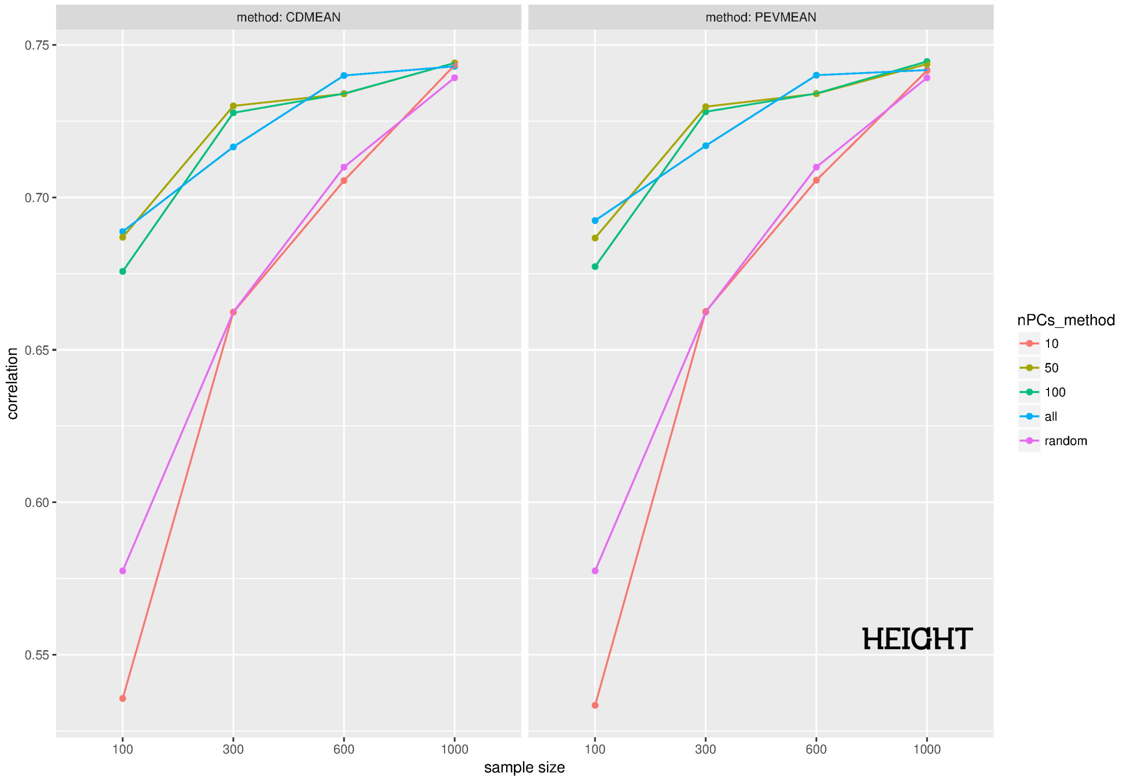


**14.** Figure S14. Degree of sacrificed accuracy using the approximations based on the use of principal components. CDMEAN and PEVMEAN based on mixed models are compared with their approximations based on ridge regression based approximations using 10, 50 and 100 principal components for the Dataset 1 and trait height.

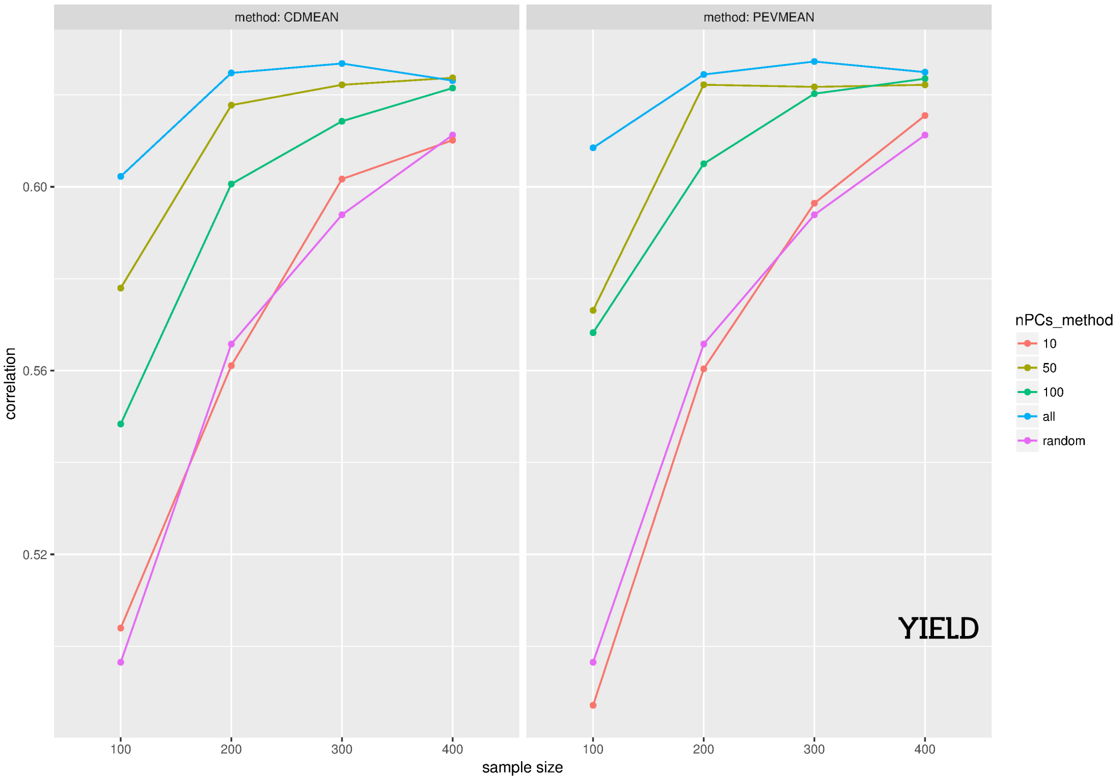


**15.** Figure S15. Degree of sacrificed accuracy using the approximations based on the use of principal components. CDMEAN and PEVMEAN based on mixed models are compared with their approximations based on ridge regression based approximations using 10, 50 and 100 principal components for the Dataset 2 and trait yield.

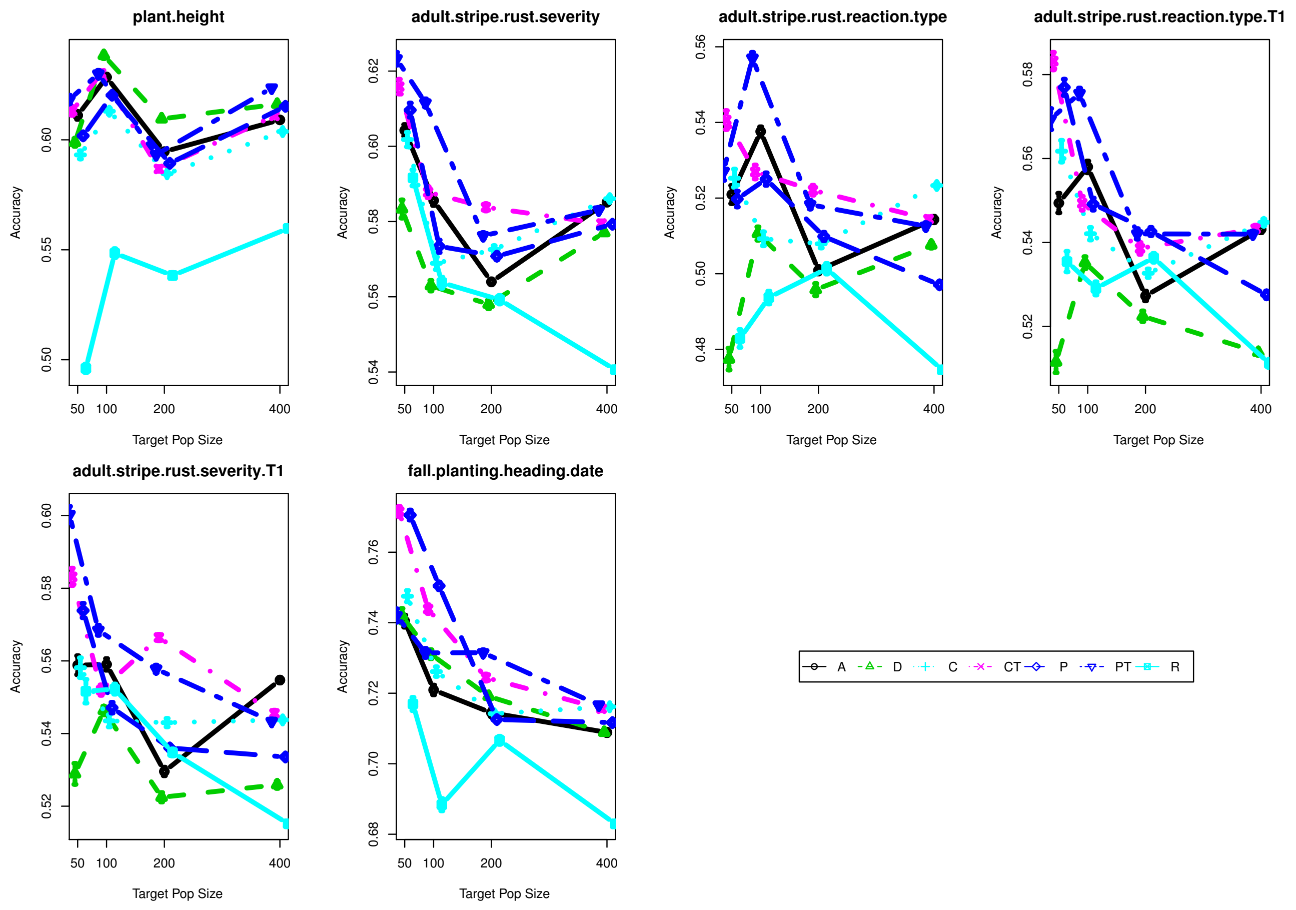


**16.** Figure S16. Effect of increasing target size for dataset 1, fixed training population size at 100 individuals. A: AOPT, D: DOPT, C: CDMEAN, CT: CDMEAN Targeted, P: PEVMean, PT: PEVMEAN Targeted, R: Random. In general, the effect of increasing the target population size is to decrease the advantages from selecting an optimized targeted training population against an optimized untargeted training populations. However, the optimized training populations retain their advantage over the random training populations.

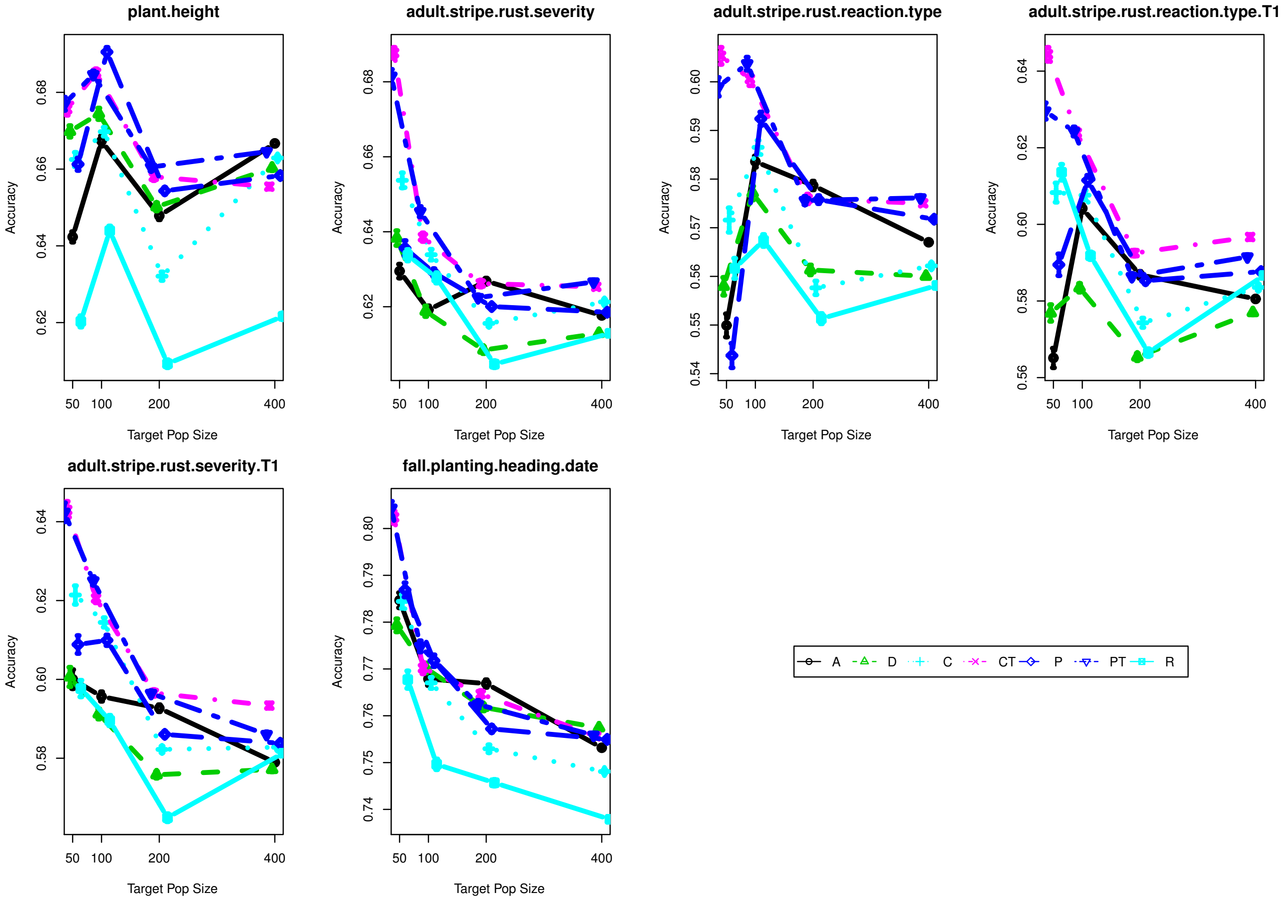


**17.** Figure S17. Effect of increasing target size for dataset 1 fixed training population size at 300 individuals.A: AOPT, D: DOPT, C: CDMEAN, CT: CDMEAN Targeted, P: PEVMean, PT: PEVMEAN Targeted, R: Random. In general, the effect of increasing the target population size is to decrease the advantages from selecting an optimized targeted training population against an optimized untargeted training populations. However, the optimized training populations retain their advantage over the random training populations.

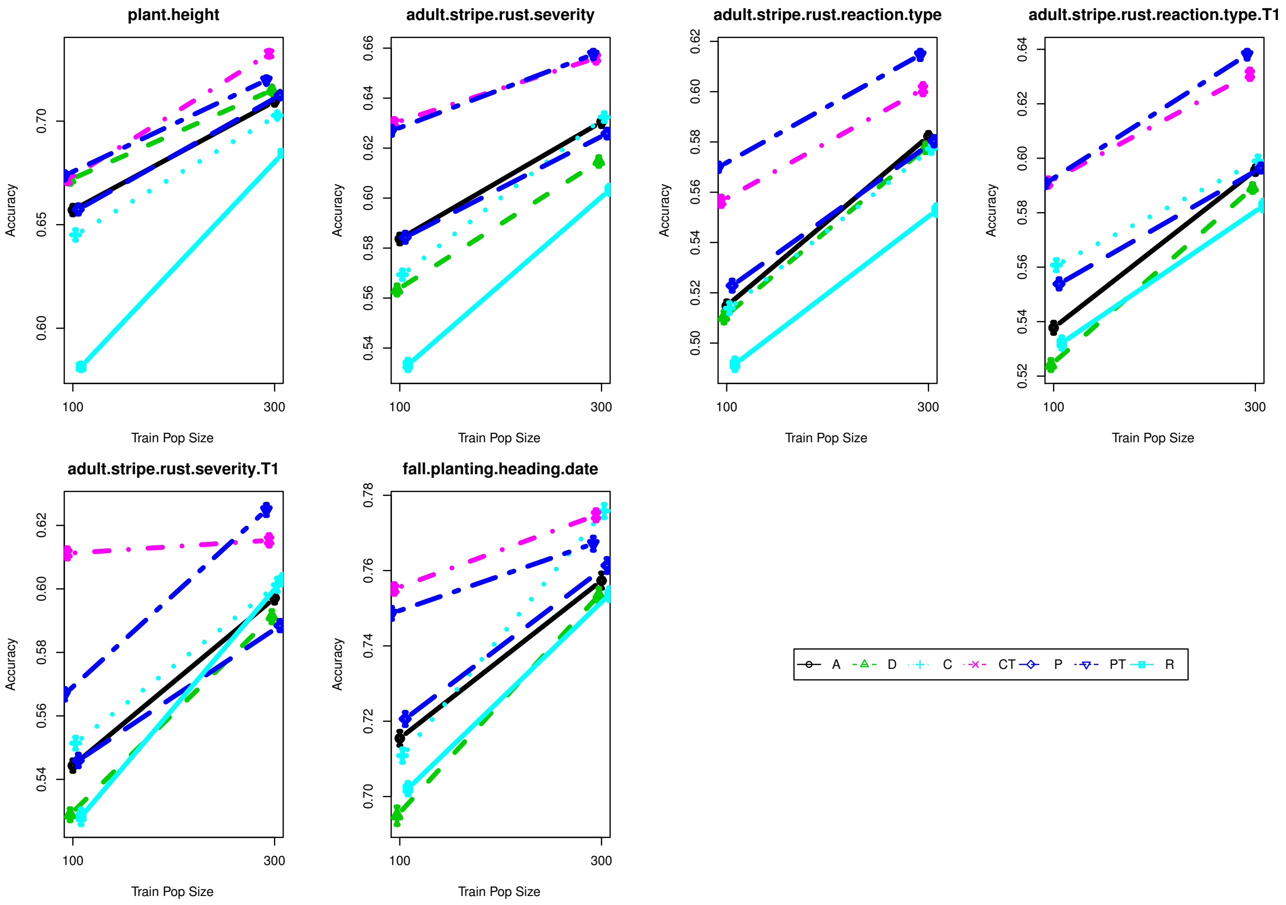


**18.** Figure S18. Dataset 1. Estimating a test population that is targeted. Target size 50. A: AOPT, D: DOPT, C: CDMEAN, CT: CDMEAN Targeted, P: PEVMean, PT: PEVMEAN Targeted, R: Random. The optimized targeted populations have an advantage over the optimized untargeted populations although the difference is decreasing as the training size increases.

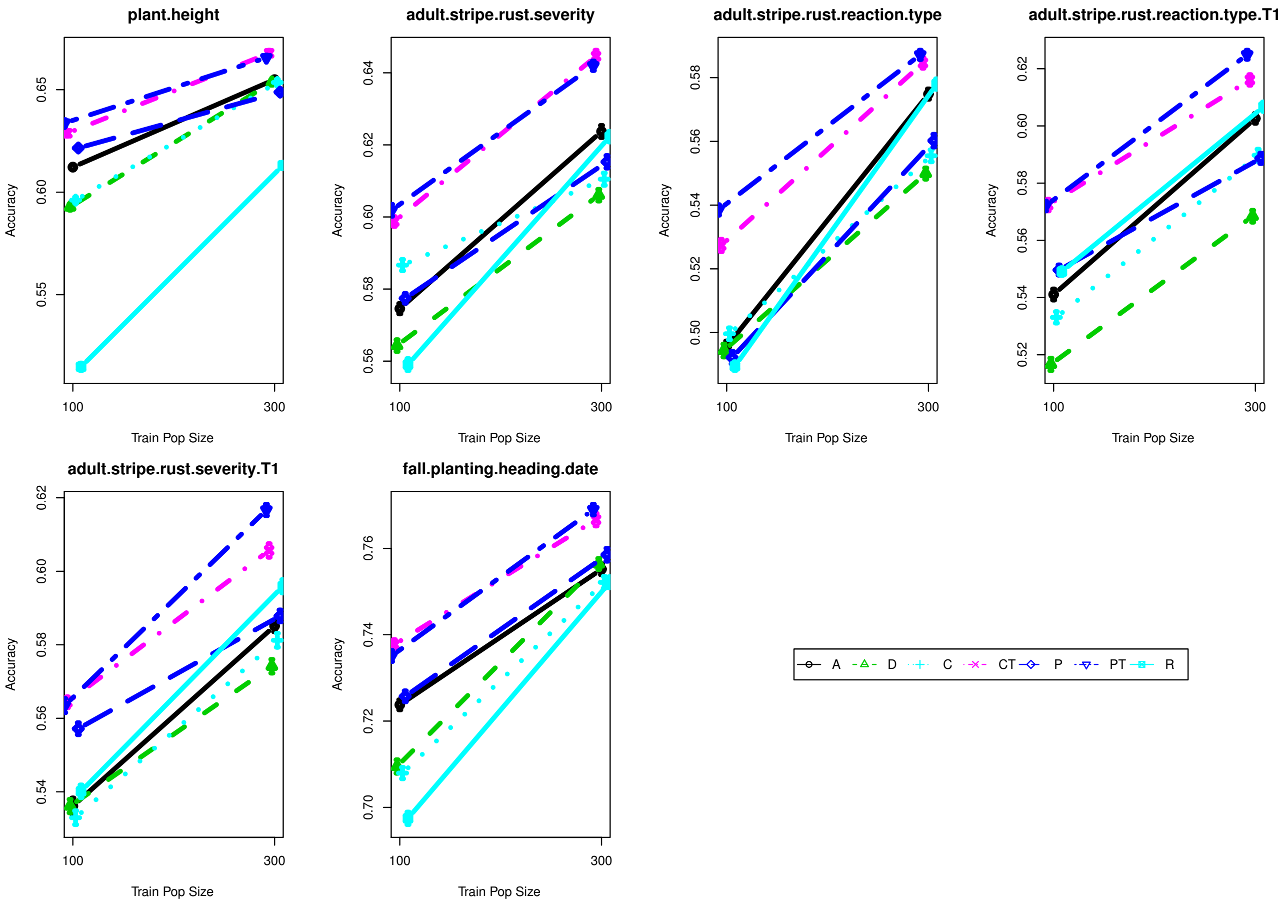


**19.** Figure S19. Dataset 1. Estimating a test population other than the one that is targeted. **Target size 50.** A: AOPT, D: DOPT, C: CDMEAN, CT: CDMEAN Targeted, P: PEVMean, PT: PEVMEAN Targeted, R: Random. The optimized targeted and the optimized untargeted training populations perform similarly for predicting a population other than the one that is targeted.

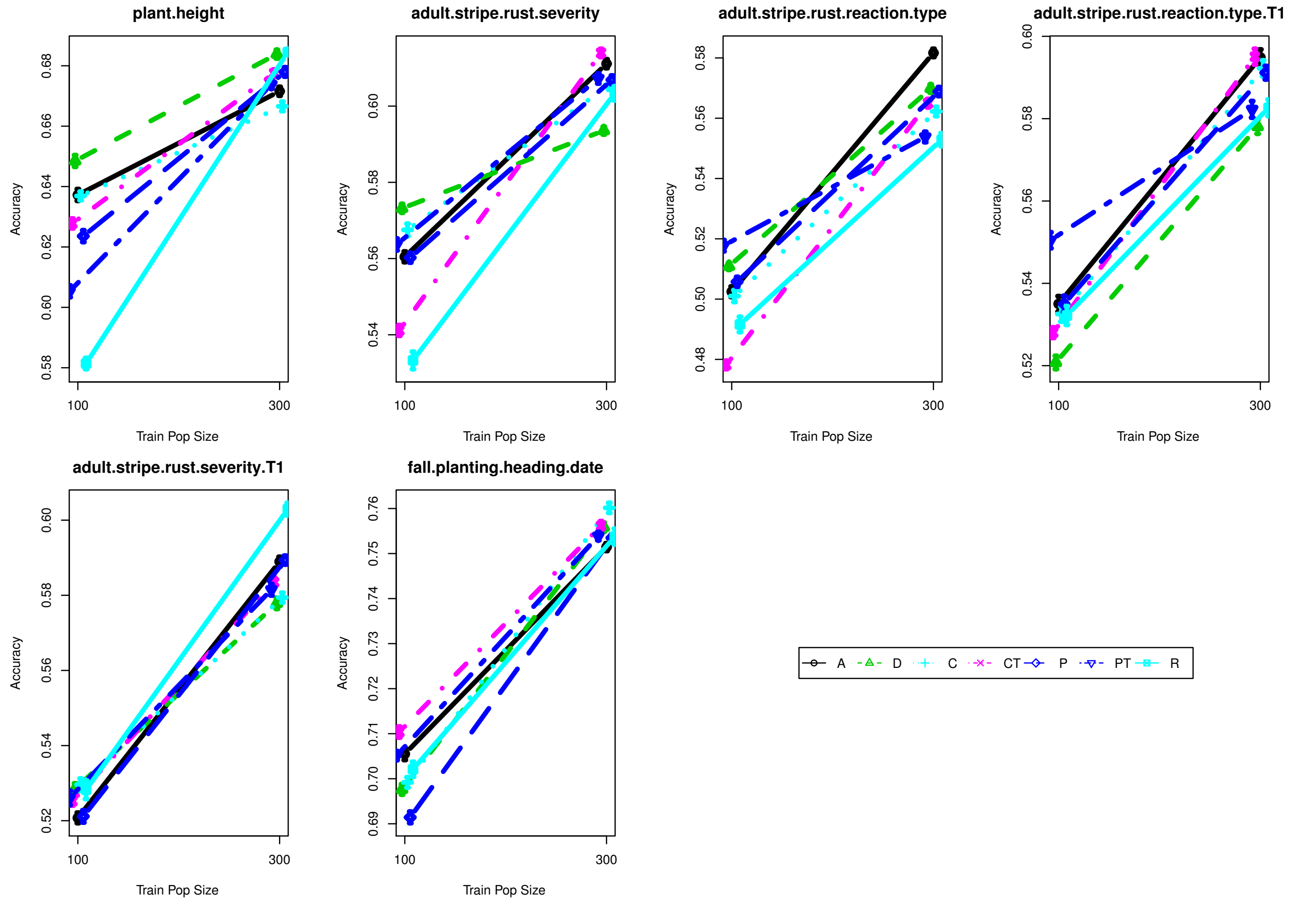


**20.** Figure S20. Dataset 1. Estimating a test population that is targeted. **Target size 100.** A: AOPT, D: DOPT, C: CDMEAN, CT: CDMEAN Targeted, P: PEVMean, PT: PEVMEAN Targeted, R: Random. The optimized targeted populations have an advantage over the optimized untargeted populations although the difference is decreasing as the training size increases. As compared to a target population size of 50, the differences between the targeted and untargeted approaches are smaller.

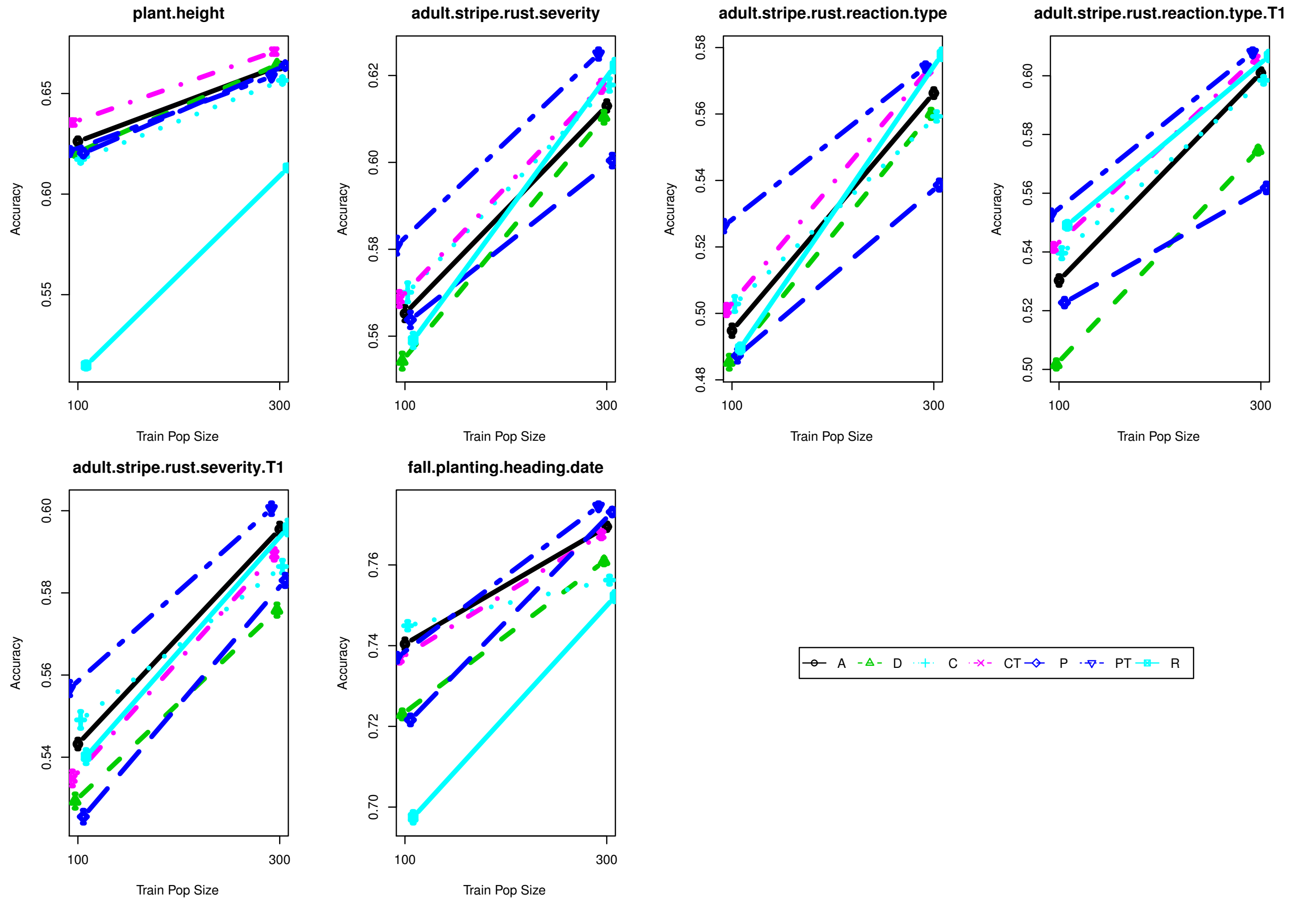


**21.** Figure S21. Dataset 1. Estimating a test population other than the one that is targeted. **Target size 100.** A: AOPT, D: DOPT, C: CDMEAN, CT: CDMEAN Targeted, P: PEVMean, PT: PEVMEAN Targeted, R: Random. The optimized targeted and the optimized untargeted training populations perform similarly for predicting a population other than the one that is targeted.
